
JOURNAL INFORMATION
==============================
NLM Title Abbreviation: Innov Surg Sci
Journal ID: Innov Surg Sci
NLM Journal ID: 101708165
Journal ID: iss

Innovative Surgical Sciences

EISSN: 2364-7485
Publisher: De Gruyter

ARTICLE INFORMATION
==============================
PMCID: PMC11917187
PMID: 40110100
DOI: 10.1515/iss-2025-2002
Article ID: iss-2025-2002
Article version: 1
Subjects: Article

Abstracts DGCH

Electronic publication date: 2025 Mar 19
Publication date: 2025 Mar
Volume: 10
Issue: Suppl1
First page: 40
Last page: 112
Copyright: © 2025 by Walter de Gruyter Berlin/Boston
Copyright year: 2025
Copyright holder: De Gruyter
License: This work is licensed under the Creative Commons Attribution 4.0 International License.
License URL: https://creativecommons.org/licenses/by/4.0/

Page count: 73

==============================

Abstracts – DCK 2025 – 142nd Congress of the German Society of Surgery – Munich, March 26–28 • DOI 10.1515/iss-2025-2002
Innov Surg Sci 2025; 10 (Special Suppl 1): s40–s112

**Multidisciplinary treatment of aorto-esophageal fistula – Developments within the last decade**
ID: 300

**Kategorie: DGCH - Chirurgische Innovationen**

Felix Strobl[1 2], Jan Stana[1], Agnes Böhm[3], Aldin Mehmedovic[1], Maximilian Pichlmaier[4], Martin Angele[5], Nikolaos Tsilimparis[1]

[1]*LMU Klinikum Campus Großhadern, Klinik für Gefäßchirurgie, München, Deutschland*, [2]*Charité Campus Benjamin Franklin, Klinik für Gefäßchirurgie, Berlin, Deutschland*, [3]*Charité Universitätsmedizin, Chirurgische Klinik CCM|CVK, Berlin, Deutschland*, [4]*LMU Klinikum Campus Großhadern, Klinik für Herzchirurgie, München, Deutschland*, [5]*LMU Klinikum Campus Großhadern, Klinik für AVT-Chirurgie, München, Deutschland*

**Background:**
Lore Although rare, aorto-esophageal fistula (AEF) remains one of the most critical diseases in cardiovascular surgery. With thoracic endovascular aortic repair (TEVAR) establishing itself as a standard treatment for pathologies of the descending aorta, the number AEF cases manifesting secondarily is rising. The lack of prospective studies or even large case series leads to an absence of evidence based therapeutic concepts.

**Materials and methods:**
We conducted a retrospective analysis of all patients treated at the University Hospital of Munich LMU for aorto-esophageal fistula (AEF) between September 2014 and May 2023 (n = 10). Additionally, a systematic search was conducted for all studies researching treatment of AEF including ≥ 5 patients and published within the last 10 years.

**Results:**
In the LMU patient-collective 40 % of manifested as primary AEF (due to thoracoabdominal aneurysm, esophageal cancer), while 60 % occurred secondary to previous TEVAR. Mean duration to manifestation post-TEVAR was 20.1 ± 5.6 months. Short term mortality within 30 days across the whole collective was 40 % (due to acute bleeding, fulminant sepsis). Initial treatment of the aorta consisted of rifampicin soaked TEVAR or TEVAR-relining in n = 7 cases, followed by bovine open aortic replacement (n = 1) or bovine patchrepair (n = 1) when viable. When possible, treatment of the esophagus consisted of primary suture (n = 1) or esophagectomy (n = 5) with gastric pull-up or colon interposition. Patients receiving esophagectomy or esophageal repair exhibited significantly longer survival (12.8 ± 4.7 vs 0.35 ± 1.3 months, p = 0.019). Across the reviewed literature, the strongest effect on survival seems to originate from surgical treatment of the esophagus, however, some groups report best single outcomes using maximally aggressive surgical treatment of both esophagus and aorta. Specific surgical strategies as well as patient characteristics vary widely.

**Conclusion:**
We found TEVAR effective in stabilizing the initial symptoms and viable as definitive aortal treatment with acceptable mid-term outcomes. Short interval esophagectomy seems to improve outcome by mitigating prothesis infection and should be considered in all patients. Open aortic replacement with stent removal should be performed when viable with respect to patient comorbidities. Interventional treatment options alone do not appear to be sufficient.

Open Access. © 2025 The Author(s), published by De Gruyter. This work is licensed under the Creative Commons Attribution 4.0 International License.

Abstracts – DCK 2025 – 142nd Congress of the German Society of Surgery – Munich, March 26–28 • DOI 10.1515/iss-2025-2002
Innov Surg Sci 2025; 10 (Special Suppl 1): s40–s112

**Biological conduits based on spider silk- A new perspective for reconstruction of extended nerve defects**
ID: 351

**Kategorie: DGCH - Chirurgische Innovationen**

Peter M. Vogt[1], Khaled Dastagir[1], Christine Radtke[2], Birgit Weyand[1], Sarah Strauß[1]

[1]*Medizinische Hochschule Hannover, Klinik für Plastische, Ästhetische, Hand- und Wiederherstellungschirurgie, Hannover, Deutschland*, [2]*Med.Universität Wien, Klinik für Plastische, Ästhetische und Rekonstruktive Chirurgie, Wien, Österreich*

**Background:**
The availability of appropriate conduits remains an obstacle for successful reconstruction of long-distance nerve defects. In previous sheep trials, we were able to bridge 6 cm nerve gaps with nerve conduits based on spider silk fibers with full functional outcomes. Here, we describe the first application of spider silk for nerve repair in humans.

**Materials and methods:**
6 patients with extended nerve defects of up to 20 cm underwent nerve reconstruction by interposition of conduits that were composed of spider silk fibers contained in autologous veins. The longitudinal luminal fibers (approx. 2500 fibers per graft) consisted of drag line silk from *Trichonephila* spiders. 6 patients were evaluated between 1 and 10 years post reconstruction, clinically, and by neurography.

**Results:**
In all patients, primary wound healing and no adverse reactions to the implanted spider silk material were observed. Patients regained the following relevant functions: protective sensibility, full flexor function with near-normal grasp and powerful function after microvascular gracilis muscle transfer, and key grip function and gross finger flexion after additional tenodesis. One patient with sciatic nerve reconstruction developed protective sensibility of the lower leg, foot, and gait, enabling normal walking and jogging. Two patients with brachial plexus injuries showed moving Tinel signs in the repaired nerve fascicles. No neuroma formation or neuropathic or chronic pain occurred in any of the patients.

**Conclusion:**
For patients with extended peripheral nerve defects in the extremities, use of conduits based on spider silk fibers offers the possibility of restoring sensory function and protection from neuroma. This kind of nerve bridges provides new perspectives for the reconstruction of complex and long-distance nerve defects.

**Clinical Application of Pancreatectomy with In-Situ Pancreatic Duct Repair and Reconstruction for Tumors of the Pancreatic Neck and Body**
ID: 435

**Kategorie: DGCH - Chirurgische Innovationen**

Gang Wang[1], Qikai Sun[2], Yajun Wei[1], Cheng Wang[1]

[1]*Department of Biliary and Pancreatic Surgery, The First Affiliated Hospital of USTC, Division of Life Sciences and Medicine, University of Science and Technology of China, Hefei, China*, [2]*Department of General Surgery, The First Affiliated Hospital of Anhui Medical University, Hefei, China*

**Background:**
Restrictive pancreatectomy with in-situ pancreatic duct repair and reconstruction offers a potential organ-preserving surgical approach for benign, borderline, or low-grade malignant tumors of the pancreatic neck and body. This study aims to investigate the feasibility and safety of this procedure in selected patients.

**Materials and methods:**
The clinical data of 3 patients admitted to the Department of Biliary and Pancreatic Surgery of the First Affiliated Hospital of University of Science and Technology of China underwent restrictive pancreatectomy plus repair and reconstruction of pancreatic duct in-situ from January to June 2023 were retrospectively analyzed.

**Results:**
Of the three patients (1 male, 2 female, aged 41-59), one case required conversion to laparotomy, while two were successfully managed with laparoscopic surgery. Intraoperative and postoperative findings confirmed benign or low-grade malignant tumors in all cases. Tumor size ranged from 1.2 to 4.5 cm, with resected pancreatic tissue lengths of 2.5 to 4.5 cm. The median operative time was 275-340 minutes, with minimal intraoperative blood loss (50-100 mL) and no need for blood transfusions. Postoperatively, all patients developed grade B pancreatic fistulas but experienced no other complications. The median time to gastrointestinal recovery was 1-4 days, and the initiation of feeding occurred by day 6-8. Hospital discharge occurred between days 10-20, with a median hospitalization cost of 36,400-55,300 yuan. Follow-up at 390-500 days showed favorable recovery with no new-onset diabetes or significant digestive dysfunction such as steatorrhea.

**Conclusion:**
Restrictive pancreatectomy combined with in-situ pancreatic duct repair and reconstruction is a feasible and safe approach for benign and borderline, and low-grade malignant tumors of the neck and body of the pancreas.

Abstracts – DCK 2025 – 142nd Congress of the German Society of Surgery – Munich, March 26–28 • DOI 10.1515/iss-2025-2002
Innov Surg Sci 2025; 10 (Special Suppl 1): s40–s112

### Evaluation of Mixed Reality Applications Using RGB Lighting

ID: 486

**Kategorie: DGCH - Chirurgische Innovationen**

Verena Uslar[1], Anahita Kesh[1], Dirk Weyhe[1], Daniela Salzmann[2], Navid Tabriz[1]

[1]*Carl von Ossietzky Universität Oldenburg, Universitätsklinik für Viszeralchirurgie, Pius-Hospital, Universitätsmedizin Oldenburg, Oldenburg, Deutschland*, [2]*apoQlar, Hamburg, Deutschland*

**Background:**
Mixed Reality (MR) overlays virtual information on real environments and can assist surgeons in precise localization of surgical landmarks and intraoperative navigation. However, the visibility of the holograms reportedly changes depending on the environmental lighting. So-called RGB lighting (red-green-blue) is already available in modern operating rooms, raising the question of which color setting is most useful for the use of MR applications. The study evaluated different lighting conditions for the visual perception of various types of holograms in surgical environments regarding the preferred combination of light and hologram.

**Materials and methods:**
Twenty participants were recruited, including surgeons, surgical assistants, and medical students. They completed a questionnaire under various lighting conditions (daylight equivalent, red, green, and blue light) while interacting with three types of holograms: volume rendering of a black/white CT, photorealistic natural rendering, and color-segmented 3D models. The questionnaire asked about the perceived brightness and contrast of the holograms under each lighting condition. Furthermore, sociodemographic data and information on prior experience with MR were collected.

**Results:**
Both the hologram type and the lighting conditions significantly influenced participants' perceived brightness ($p < 0.05$). Green light was perceived as the brightest, while blue light appeared the darkest. This also corresponds to the objective lux measurements. Significant differences were also observed in the perceived contrast between the lighting conditions ($p < 0.05$), with blue light offering the best contrast (see Fig. 1).

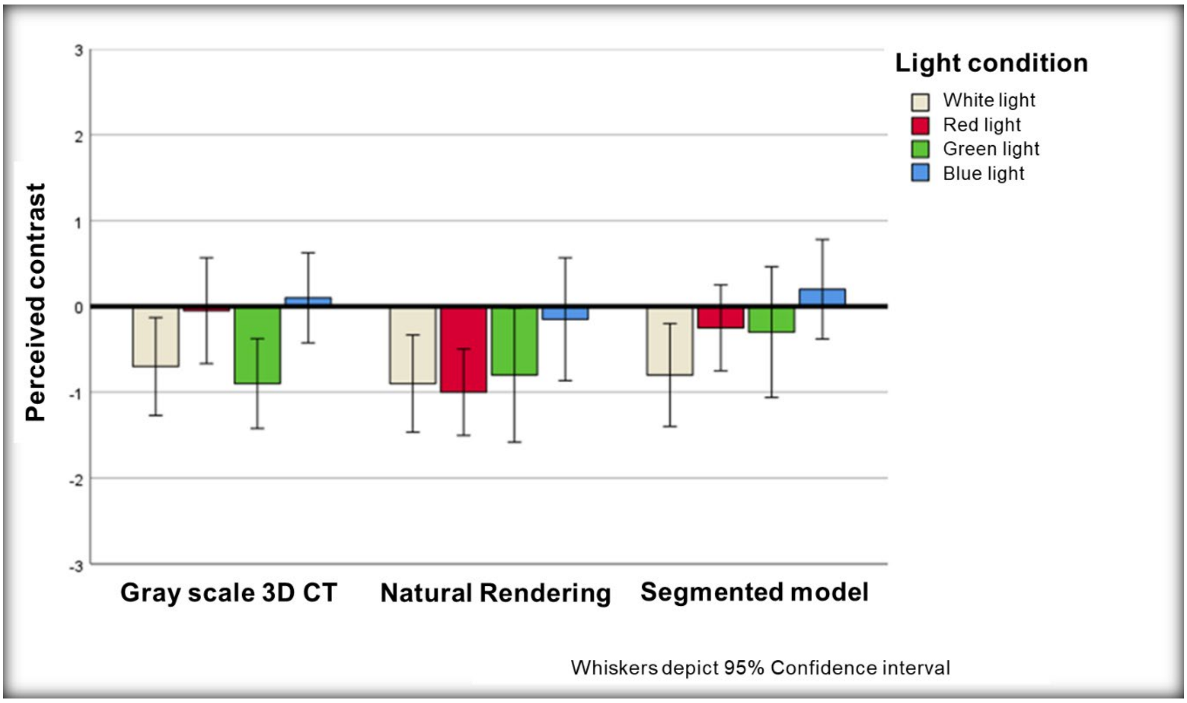

Fig. 2: Perceived contrast depending on light condition and hologram type; 0 = ideal contrast; negative values: too little contrast; positive values: too contrast-rich

**Conclusion:**
This study suggests that certain lighting conditions may be better suited for optimal virtual hologram visualization, considering factors like brightness and contrast. However, individual preferences and application contexts may also play a role in the hologram design. Future research could examine additional lighting spectra and real surgical conditions to validate these results. In summary, this study emphasizes the importance of lighting conditions for enhancing the visual perception of AR holograms in surgery. By understanding the interplay between lighting and hologram characteristics, surgeons can optimize visualization to achieve better surgical outcomes.

**Between General Ward and Intermediate Care: A step-wise approach to perioperative care**
ID: 605

**Kategorie: DGCH - Chirurgische Innovationen**

Henrik Nienhüser[1], Niels Siegel[1], Christopher Hampel[2], Birgit Trierweiler-Hauke[2], Christoph Michalski[1], Markus Mieth[1]

[1]*University Hospital Heidelberg, Department of General, Visceral and Transplantation Surgery, Heidelberg, Deutschland*, [2]*University Hospital Heidelberg, Heidelberg, Deutschland*

**Background:**
Postoperative intensive care has changed dramatically over the past two decades. On the one hand, there has been a steady shift toward outpatient procedures and a steady reduction in postoperative hospital stay. This has been achieved by minimally invasive surgical procedures, improved anesthesia and the introduction of ERAS protocols. On the other hand, perioperative therapy after major abdominal surgery has evolved to become an integral part of major oncologic resection or organ transplantation. This evolution has led to a significant reduction in perioperative morbidity and mortality. However, this treatment requires adequate resources, and the distribution of available intensive care capacity often represents a relevant conflict, leading to case delays and patient dissatisfaction. Historically, postoperative care has been provided either in intensive care units or on general wards. As this dichotomous separation has several limitations, many hospitals have introduced Intermediate Care Units (IMC) with a special focus on patients requiring "limited" intensive care.

**Materials and methods:**
A highly specialized intermediate care unit for visceral surgery was introduced at Heidelberg University Hospital in 2004, which is essential for the post-operative care of patients in the hospital. Due to the ongoing shortage of ICU capacity and the discrepancy between nursing and medical resources on the ICU and general wards, an advanced care unit was established in October 2023 for patients who are awaiting transfer to the general ward but still require intensive monitoring or care that cannot be provided on the general ward. The goal is to optimize the transition from a critical care environment to the general ward and avoid readmissions. All patients had undergone surgery and required initial intensive/intermediate care. Advanced care was provided by IMC physicians, who are available 24 hours a day, and nurses from the general ward, where the advanced care unit is integrated. The first cohort of patients treated on this advanced care unit was analyzed, and patient characteristics and length of stay were recorded using digital patient records. SOFA score was used to assess the degree of organ dysfunction, while TISS-28 was used to quantify the intensity of therapeutic interventions for patients.

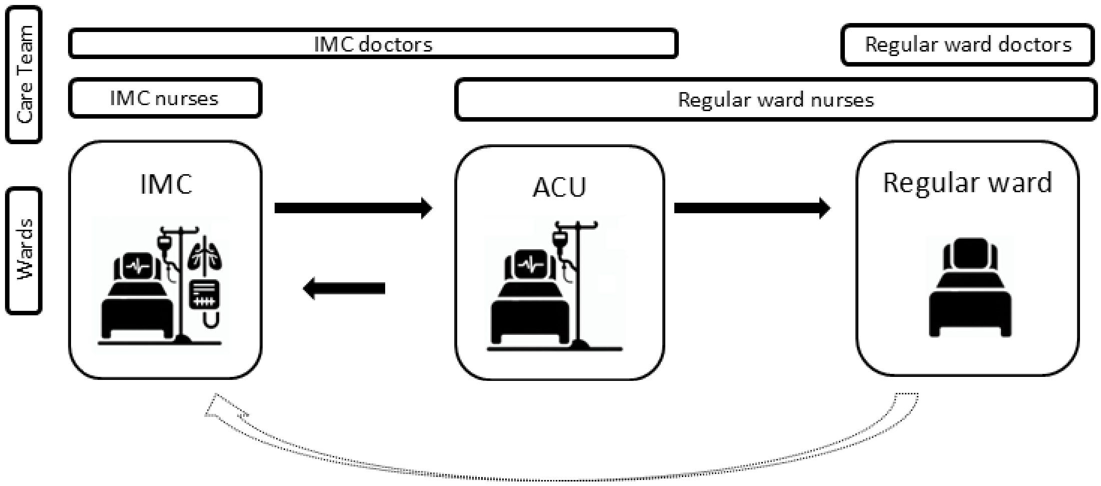

**Results:**
A total of n=110 patients were treated in the newly established advanced care unit. Median age was 63.8 (+/- 13) years and all patients underwent surgery requiring postoperative surveillance. The majority of patients underwent either pancreatic resection (n=42; 38.5%), liver resection (n=11; 10.1%) or esophageal resection (n=8; 7.3%). Median length of stay in advanced care unit was 3.8 days (+/- 4.0) and 73.6% (n=81) of patients were transferred to general ward. In contrast, 20% (n=22) of patients had to be transferred back to the intermediated care unit. Either transfer to another hospital or discharge to home accounted for the remaining 6.4% (n=7) of patients. Patients' median SOFA score the day before transfer to the advanced care unit was 1, median TISS-28 score was 21. After transfer to the general ward, patients treated in the step-down unit had a median hospital stay of 12.5 days (+/-7.8).

**Conclusion:**
To our knowledge, this is the first report of a stepwise approach to postoperative intensive care after major abdominal surgery. Given that the lack of postoperative intensive care limits the potential surgical therapy options, we present a new concept of stepwise postoperative care. The SOFA score of the patients prior to referral to the advanced care unit indicates no septic state, but the TISS-28 score still indicates a moderate level of therapeutic intervention required. The majority of patients were able to leave the advanced care unit after a short stay and were transferred to the general ward. Some patients had to be transferred back to the IMC, indicating that they weren't ready for the general ward, which is easier to facilitate with the same team.

**Teleneurosurgery between Greenland and Denmark**
ID: 831

**Kategorie: DGCH - Chirurgische Innovationen**

Najannguaq Jørgensen[1], Simon Bernth-Andersen[1], Frank Damborg[1], Thorbjørn Søren Rønn Jensen[2], Anders Vedel Holst[2], Kåre Fugleholm[2], Ramona Åstrand[2], Alexander Lilja-Cyron[2], David Kulber[3], Kylie Tanabe[3], Marianne Juhler[2], Tiit Mathiesen[2], Torstein Meling[2], Jeppe Lohfert Haslund-Vinding[2]

[1]Department of Surgery, Dronning Ingridip Napparsimavissua, Nuuk, Greenland; [2]Department of Neurosurgery, The Neuroscience Centre, Copenhagen University Hospital Rigshospitalet, Copenhagen, Denmark; [3]Department of Orthopedic Surgery, Cedars Sinai Medical Center, Los Angeles, United States of America

**Background:**
The Department of Neurosurgery at the University Hospital of Copenhagen Rigshospitalet is responsible for tertiary care of patients from Greenland. Due to geographical remoteness and weather-related logistical challenges, ideals of equal-healthcare including timely access to neurosurgical interventions is often difficult to achieve. This article describes a new initiative exploring the role of online supervision technology to allow neurosurgical care in remote areas.

**Materials and methods:**
In 2024, a teleneurosurgical collaboration involving surgeons from Queen Ingrid's Hospital in Nuuk, Greenland and Copenhagen University Hospital Rigshospitalet, Denmark was initiated. The program included training local surgeons to perform cranial neurosurgical procedures using smart glasses, enabling real-time supervision from neurosurgeons at Rigshospitalet.

**Results:**
The first patient operated with teleneurosurgical supervision was successfully operated in May 2024.

**Conclusion:**
Real-time visualization and interactive communication between the surgeon at Queens Ingrid’s Hospital, Nuuk, Greenland and the neurosurgeon in Copenhagen was technically satisfactory providing clear transmission and valuable interactive features such as drawing and zooming for precise guidance.

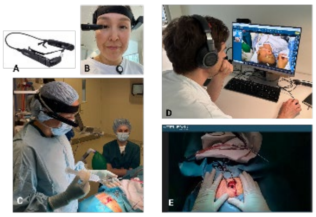

### Outcome on Limb-level in Peripheral Arterial Disease by location of Atherosclerotic lower limb lesions: An observational study from a high-volume Center

ID: 596

**Kategorie: DGCH - Chirurgische Versorgung von Menschen in prekären Lebenssituationen**

Anne Zimmermann[1], Daniel Seehofer[1], Daniela Branzan[2], Konstantin Lukas Uttinger[1]

[1]*Universitätsklinikum Leipzig, Leipzig, Deutschland*, [2]*Gefäßmedizin Technische Universität München, München, Deutschland*

**Background:**
Peripheral Arterial Disease (PAD) accounts for a major disease burden worldwide and is associated with high risk of morbidity and mortality, especially due to cardiovascular and limb events. It is known that different PAD patterns as to the affection of different vessels exist. It is unclear how this, on a limb level, affects long-term limb-associated outcome, i.e. major limb events.

**Materials and methods:**
This is a retrospective single-center analysis of all adult patients with PAD as main ICD diagnosis (I70.2) admitted to the University Hospital Leipzig between January 1st 2018 and December 31st 2021, including readmissions. Patients were followed up as to their life status until November 30th 2023 using an electronic database in collaboration with the official German registry office on patient level. On a limb level, the location of atherosclerotic lesions was allocated to "inguinal, subinguinal, infrapoplitea, 2 of 3, 3 of 3" based on available angiographies, which is referred to as primary lesion. On limb level, the primary endpoint was occurrence of major limb events (defined as any amputation above the ankle or a major limb re-revascularisation, i.e. surgical revascularisation after primary revascularisation).

**Results:**
2,067 individual patients were analysed, equaling 3,916 lower limbs due to 183 admissions with a coded previous amputation surgery. Age at first admission was 68 in median (interquartile range, IQR, 61-78), and 595 patients were female (28.8%). The majority of PAD Fontaine stages at first admission were coded as IIb in 44.9% (926 patients) and as IV in 35.4% (729 patients). In 45.5% (941 patients), diabetes mellitus was coded as side diagnosis. On limb level, in 1,663 cases (42.5%), no allocation of atherosclerotic lesions was possible due to missing angiographies. In 304 cases (7.8%), the primary lesion was inguinal, while it was subinguinal in 482 limbs (12.3%) and infrapopliteal in 299 limbs (7.6%). In 988 cases (25.2%), two levels were affected, and in 180 cases (4.6%), lesions in all three levels were present. On limb level, occurrence of major limb events was 4.2% overall. Occurrence of major limb events differed by primary lesion, i.e. was lowest in case of isolated inguinals lesions (2.6%) and was highest (12.2%) in case of stenoses in three of three locations. In reference to the primary lesion being found in all three locations, in a multivariable Cox-regression adjusting for primary revascularisation, age, sex, diabetes mellitus and frailty, the risk of a major limb event was decreased in case of inguinal, subinguinal, and infrapopliteal stenoses (HR 0.26, 95% CI 0.13-0.51, p<.001, HR 0.26, 95% CI 0.11-0.62, p=.002, HR 0.58, 95% CI 0.34-0.98, p=.041, respectively), and was equally likely case of lesions in two of three locations.

**Conclusion:**
On a limb level, PAD patterns were associated with a stratified risk of limb events: the highest risk of a major limb event, i.e. any amputation above the ankle or a surgical limb re-revascularisation after primary revascularisation, was found in case all three or two of three locations (inguinal, subinguinal, and infrapopliteal) were affected in the respective limb.

### Global, regional, and national burden of pancreatic cancer and its risk factors from 1990 to 2021: a cross-sectional study for the Global Burden of Disease Study 2021

ID: 430

**Kategorie: DGCH - Globale Chirurgie**

Yajun Wei[1], Gang Wang[1], Cheng Wang[1]

[1]*Department of Biliary and Pancreatic Surgery, The First Affiliated Hospital of USTC, Division of Life Sciences and Medicine, University of Science and Technology of China, Hefei, China*

**Background:**
The increasing global incidence of pancreatic cancer necessitates up-to-date data to improve screening, early detection, treatment strategies, and resource allocation. This study aims to analyze global trends in pancreatic cancer incidence and mortality, and identify key risk factors across different regions and demographics.

**Materials and methods:**
Pancreatic cancer incidence and mortality estimates were derived from the Global Burden of Disease (GBD) 2021 study, categorized by age, sex, and location. The cause of death ensemble model was employed to calculate global age-standardized incidence and mortality rates over the study period.

**Results:**
The global age-standardized incidence of pancreatic cancer increased 2.45-fold, from 207,905 cases in 1990 to 508,532 cases in 2021, with a 2.39-fold rise in deaths. The percentage change in global age-standardized incidence rate from 1990 to 2021 was 0.089 (95% CI: 0.003–0.171), while for mortality, it was 0.052 (95% CI: -0.033–0.131). The highest increases in incidence and mortality in 2021 occurred in Greenland, Monaco, and Uruguay. Males had higher rates than females, with the greatest burden seen in the 70-74 age group. Major global risk factors in 2021 included high body mass index, elevated fasting plasma glucose, and tobacco use.

**Conclusion:**
Pancreatic cancer incidence and mortality remain significantly high, especially in high Socio-Demographic Index (SDI) regions. Addressing modifiable risk factors such as high body mass index, elevated fasting plasma glucose, and tobacco use is essential for reducing the global burden of pancreatic cancer. Targeted prevention and control strategies should be prioritized to mitigate these risks.

**assessment of global surgery understanding in the european region**
ID: 491

**Kategorie: DGCH - Globale Chirurgie**

Dina Tadros[1], Judith Lindert[2], Nicolo Marchesini[3]

[1]*Universitätskinderspital Zürich, Zürich, Schweiz*, [2]*Universitätsklinikum Rostock, Rostock, Deutschland*, [3]*Azienda Ospedaliera Universitaria Integrata di Verona, Neurosurgery, Verona, Italien*

**Background:**
Surgical care plays an essential role in addressing a wide range of health conditions, including trauma, cancer, maternal and child health, and even some infectious diseases. Despite this, Global Surgery (GS) remains underappreciated as an integral part of a well-functioning health system.
Over the past decade, recognition of the importance of GS within international health agendas has grown. While Europe is home to some of the most advanced healthcare systems globally, its role in addressing global disparities in surgical care has been inconsistent, lacking sustainability and effectiveness.
Understanding the current level of GS awareness among healthcare professionals in Europe is essential for identifying gaps, informing policy decisions, and improving surgical care delivery. This study aims to assess awareness and perceptions of GS in Europe through a comprehensive survey, providing insights into the factors that influence surgical accessibility and quality.

**Materials and methods:**
A survey was designed to evaluate awareness and understanding of Global Surgery among healthcare professionals across Europe. The survey follows the Cross-Cultural Survey Guidelines (CCSG) of the Comparative Survey Design and Implementation (CSDI) Guidelines Initiative.
Europe was defined as the European Region according to the World Health Organization (WHO).
The online survey was disseminated across professional surgical societies, GS working groups, and the public using social media channels and email via the snowballing method.

**Results:**
Between 05/23 and 09/24 100 responses were collected. The majority of the respondents come from high-income countries within the European Region (Germany, the UK, Switzerland, the Netherlands). Almost half of the respondents were specialist physicians, with a mean age of 36-45 years, while the second-largest group of respondents were residents. A total of 55.56% of respondents agreed that there are already enough opportunities available to participate in Global Surgery work. Improving the health conditions of people in the host country was cited as the primary motivation for engaging in Global Surgery by 87.30% of respondents. However, a lack of support from their current workplace was considered "very relevant" (31.75%) and a lack of time "relevant" (41.30%) obstacles to participating in Global Surgery activities. Of the 70.91% of respondents who had previously been involved in Global Surgery activities, 42.55% did so through a non-governmental organization (NGO), primarily in clinical care (79.17%). More than half of the respondents had been involved in activities lasting less than four weeks (51.06%). Among those who participated in Global Surgery work, 62.50% were trained on the job, while 47.92% had completed an official preparatory course before their engagement.
When asked about the experiences they were unprepared for during their involvement, some of the open-ended responses included: Lack of “cultural and social role models for women”, “surgical expertise, lack of resources in the local hospitals, lack of surgeons in the local health system”, “should have been trained in other surgical fields before”, “better mentoring with a mentor with good global surgery experiences”, Understanding that the practice of Global Surgery is different from the clinical practice of surgery. , “Intercultural challenges”, “ I didn´t know about the health system, health professionals”, “lack of training for clinical care in an LMIC context” , and “students having to fulfill professional roles”.

**Conclusion:**
These responses indicate that many of the survey respondents were clinicians who had been involved in clinical placements abroad—mainly short-term missions. Previous research suggests that this is a widespread understanding of what Global Surgery entails. As such, our study aimed to explore some of the issues surrounding involvement in short-term missions, which may have introduced a pre-existing bias that influenced respondents' perceptions.
Many respondents lacked a comprehensive understanding of the complexity of Global Surgery, focusing primarily on the clinical aspect. There remains a misconception that doctors trained in high-income countries should simply bring surgical expertise and knowledge to low-income countries. While the intentions are often good, they are frequently confronted with systemic disparities that they fail to fully grasp.
Europe is one of the greatest contributors to Global Surgery efforts, and there is a pressing need to enhance the knowledge about the discipline's complexity. This can help guide political involvement and improve NGO work in a more structured manner. This survey can inform political decision-making and shape training and educational efforts in surgical residencies and medical schools.

### Holding the Edge: Retention of Laparoscopic Skills in Novices after Basic Curriculum

ID: 78

**Kategorie: DGCH - Herausforderungen in der chirurgischen Weiterbildung**

Eleni Felinska[1], Benjamin Otto[1 1], Thomas Fuchs[1], Zi-Wei Chen[1], Estelle Willuth[1], Annabelle Sofia Gerhäuser[1], Beat Müller[2], Felix Nickel[3]

[1]*Heidelberg, University of Heidelberg, Department of General, Visceral and Transplant Surgery, Heidelberg, Deutschland*, [2]*Clarunis University Digestive Health Care Center Basel, Clarunis University Digestive Health Care Center Basel, Department of Surgery, Basel, Schweiz*, [3]*University Medical Center Hamburg Eppendorf, Department of General, Visceral, and Thoracic Surgery, Hamburg, Deutschland*

**Background:**
In the real world, junior surgeons complete the basic laparoscopic curriculum at various time points. The actual deployment in the operating room often occurs much later, yet the acquired skills must remain readily accessible. Therefore, it is crucial to design the training to be as efficient as possible and to ensure that skills are readily available when needed. Our study aims to investigate the retention and recall of laparoscopic skills acquired during basic training over a certain period. Additionally, we will assess the efficiency of acquiring new competencies in this setting compared to laparoscopically naïve students.

**Materials and methods:**
A total of 19 novice and 19 advanced medical students performed both basic and complex laparoscopic tasks. Skill retention and the acquisition of new laparoscopic skills were evaluated by measuring the total number of errors, time to complete the task, and through global and task-specific Objective Structured Assessments of Technical Skills (OSATS). Task load and difficulty were assessed using the NASA Task Load Index questionnaire (NASA-TLX) and a visual analog scale (VAS).

**Results:**
Advanced participants completed the PEG transfer task significantly faster than novices (177 ± 46.6 sec vs. 248.8 ± 87.6 sec, $p < 0.001$). In more complex laparoscopic tasks, such as cholecystectomy, no significant differences in OSTAS and GOALS scores were observed between groups. However, the advanced group performed better in crucial steps and showed superior knot-tying performance, requiring fewer attempts to reach proficiency (7.4 ± 4.4 vs. 11.0 ± 4.2, $p < 0.05$) and completing the task faster (240.4 ± 72.4 sec vs. 567.7 ± 278.9 sec, $p < 0.0001$). Both groups performed similarly in acquiring new skills, such as enterotomy closure.

**Conclusion:**
Skill retention was demonstrated for basic laparoscopic tasks, but not for more complex procedures such as cholecystectomy. No significant differences were observed in the acquisition of new laparoscopic skills between the novice and advanced groups. These findings suggest that while basic skills may be retained over time, more complex tasks require ongoing practice to maintain proficiency, regardless of the initial skill level.

**"YouTube" for surgical training and education in donor nephrectomy: Friend or foe?**
ID: 168

**Kategorie: DGCH - Herausforderungen in der chirurgischen Weiterbildung**

Mohamed El-Mahrouk[1], Derar Jaradat[1], Tim Eichler[1], Robert Sucher[1], Christian Margreiter[1], Andri Lederer[1], Robert Karitnig[1], Antonia Geisler[1], Nora Jahn[2], Hans Michael Hau[1]

[1]*Medizinische Universität Graz, Allgemein-, Viszeral- und Transplantationschirurgie, Graz, Österreich*, [2]*Medizinische Universität Graz, Anästhesiologie und Intensivmedizin, Graz, Österreich*

**Background:**
The COVID-19 pandemic has accelerated the shift toward e-learning and online education in surgical training. With the increasing prevalence of end-stage chronic kidney disease, kidney transplantation is in high demand. Donor safety is crucial in nephrectomy procedures, highlighting the importance of effective training. This study evaluates the quality and effectiveness of YouTube videos focusing on laparoscopic and robotic donor nephrectomy for surgical education.

**Materials and methods:**
On October 24, 2023, searches on YouTube for "laparoscopic live donor nephrectomy" and "robotic live donor nephrectomy" returned 123 videos, with 63 included in the study. Popularity was evaluated using the Video Power Index (VPI), while reliability and quality were assessed using the LAP-VEGaS Video Assessment Tool and Journal of the American Medical Association (JAMA) benchmark criteria. Additionally, a structured checklist called the "Live Donor Nephrectomy Scoring System (LDNSS)" was created to evaluate completeness and educational value of procedural steps.

**Results:**
Out of 63 videos reviewed, laparoscopic surgical procedures were depicted in 71.4% of them, while robotic approaches were shown in 28.6%. Academic backgrounds were associated with 54% of the videos, and individual physician backgrounds with 46%. Mean scores were LAP-VEGaS 9.79 +/- 3.87, VPI 6.32 +/- 3.31, and LDNSS 9.68 +/- 1.97. JAMA scores varied, with 34.9% receiving 1 point, 34.9% receiving 2 points, 17.5% receiving 3 points, and 12.7% receiving 4 points. Academic videos scored significantly higher in LAP-VEGaS and LDNSS (all $p<0.01$). While LAP-VEGaS, VPI, and LDNSS scores correlated significantly (all $p<0.05$), no correlation was found between JAMA score and other scoring systems. Videos with more clicks and likes showed significantly better scores across all measures (all $p<0.05$).

**Conclusion:**
Amidst the challenges posed by the pandemic on surgical education, YouTube emerges as a valuable resource for learning about laparoscopic and robotic donor nephrectomy for living kidney donation. However, the quality and reliability of these videos vary greatly, and many lack thorough reviews, leading to incomplete information. To enhance their educational value, it's proposed that videos undergo professional evaluation before publication and adhere to standardized, structured, and validated scoring systems, ensuring logical structure and improved quality.

**Global Experience of Surgeons in Germany - A Survey Study**
ID: 495

**Kategorie: DGCH - Herausforderungen in der chirurgischen Weiterbildung**

Hannah Bleichroth[1], Judith Lindert[2]

[1]*Abteilung für Allgemein-/Viszeralchirurgie, St. Josefskrankenhaus, Freiburg im Breisgau, Deutschland*, [2]*Klinik für Kinderchirurgie, Universitätsmedizin Rostock, Rostock, Deutschland*

**Background:**
Globally, 5 billion people lack access to safe, affordable surgical care. "Global Surgery" has been gaining importance, particularly in the U.S., in terms of training and education.
To date, there has been no systematic data collection on the number of surgeons working in Germany who have also worked abroad, nor on the conditions they face. There is also a lack of data regarding specialist training abroad, especially in low- and middle-income countries (LMICs). To gain preliminary insights into how German surgeons prepare for and work abroad, we conducted an online survey among surgeons working in Germany.

**Materials and methods:**
We designed an online survey and distributed invitations to participate to all surgical departments of German university hospitals, as well as to all surgical societies and through established networks. Data collection occurred from May to October 2021.

**Results:**
A total of 218 usable responses were received. Among all respondents, 65% reported professional experience abroad in an LMIC, including during medical school or other professional backgrounds. A total of 34% of participants planned to work abroad within the next year, although only 7% of surgeons still in training and 11% of respondents under the age of 40 had such plans.
Eighteen respondents (8%) had completed part of their specialist training abroad. Some had training periods of up to 24 months accepted from countries such as the U.S., Canada, the UK, as well as Tanzania, Uganda, and Bulgaria. Shorter approved periods were reported for countries like Cameroon, Mexico, and Yemen. One respondent reported that their application for recognition of training abroad had been denied. Only 3 of the 18 respondents who trained abroad were under the age of 50.
Most foreign assignments were planned for a duration of less than 3 months (n=61), although 37% of respondents with work experience in an LMIC had worked abroad for more than 24 cumulative months. Most of these assignments were in African regions. Fifty-one respondents arranged unpaid leave with their German employers for their time abroad, while 42 resigned from their positions. Eighteen remained employed and received full pay, and 9 received partial pay. Only 9 respondents planned to work in public health, and 4 aimed to work in research.
In general, 84% of respondents who had worked abroad found the experience "very helpful" or "helpful" for their work in Germany. However, only about half of the respondents felt that their superiors had a positive view of their time abroad. Regarding the compatibility of working abroad with their responsibilities in Germany, 44% found it compatible, while 31% felt it was not. Seventy-five percent of German physicians who responded believed they have a global responsibility. However, only 26% had participated in any kind of Global Surgery event.

**Conclusion:**
A large proportion of surveyed surgeons had previous international experience, with most viewing it as beneficial for their work in Germany. International assignments tended to be short (<3 months), and many respondents worked in African countries. Surgeons who worked in LMICs often accumulated over 24 months of cumulative work abroad, primarily in clinical or teaching roles. Research and public health engagement in these settings was minimal, reflecting the lack of focus on surgical topics in international agendas such as the WHO’s.
Surgeons often only plan to work abroad after becoming specialists and over the age of 40, despite the fact that foreign training periods were frequently recognized. There is a lack of structures and opportunities to facilitate orientation toward global surgery. Furthermore, Global Surgery is not a prominent topic in German universities. Since 75% of respondents believe German surgeons have a global responsibility, specialist training abroad should be more accessible, and Global Surgery should be a well-known concept among all surgeons working internationally. This would enable the shared global responsibility to be shouldered together with our foreign colleagues.

### Needs and wishes of GenZ medical students – a prospective cross sectional single center study

ID: 497

**Kategorie: DGCH - Herausforderungen in der chirurgischen Weiterbildung**

Stefan Mohr[1], Elias Khajeh[2], Johanna Fellhofer-Hofer[2], Felix Nickel[3], Arianeb Mehrabi[2], Fee Klupp[2 2]

[1]*Universitätsklinikum Heidelberg, Klinik für Anästhesiologie, Heidelberg, Deutschland*, [2]*Universitätsklinikum Heidelberg, Klinik für Allgemein-, Viszeral- und Transplantationschirurgie, Heidelberg, Deutschland*, [3]*Universitätsklinikum Hamburg-Eppendorf, Klinik für Allgemein.,Viszeral - und Thoraxchirurgie, Hamburg, Deutschland*

**Background:**
Current medical students are mostly appurtenant to GenZ. Special personality traits were assigned to them, which differ from the ones of former generations like GenY, GenX or Baby Boomers. They are called digital natives, watch out for work-life balance, as well as for social responsibilities and sustainability. Therefore, we wanted to assess the motivational status as well as behavioral aspects of learning, work - life balance and career plans of fourth year medical students during their surgical semester.

**Materials and methods:**
138 fourth-year medical students participated in the study. Study participation was voluntary and anonymous. An evaluation questionnaire with either dichotomous response options or multiple response options was used. Evaluation questions were analyzed using a 5-point Likert scale. A p-value less than 0.05 was considered to be significant.

**Results:**
Mean age was 23 years. Asked for their personal reasons to study medicine the most evaluated answers were the desire of helping and having a save job later on. Moreover, the majority of students favor online learning tools instead of medical books. Likewise, the majority of students stated that they did not want to become a surgical specialist after their exam. The most important attributes for their work are satisfaction and fulfillment, having power or doing research are among the least favored answers. Moreover, only few students have the goal of becoming a chief physician, whereas practicing physician is worth achieving to them. And nearly the half stated that there are not enough holidays.

**Conclusion:**
A changing mindset in GenZ medical students is given with distinct visions of their individual professional future. Reconsideration and, if applicable, adaption of medical teaching methods in undergraduate students as well as in postgraduate physicians should be in mind in order to take needs and wishes of GenZ members seriously resulting in new expectations and requirements of employers and medical teachers.

**Entrusted professional activities - a paradigmshift in surgical training from quantity to quality**
ID: 539

**Kategorie: DGCH - Herausforderungen in der chirurgischen Weiterbildung**

Steffen Axt[1 1], Luis Hummel[1], Stefanie Hehl[1], Adrian Marty[2], André Mihaljevic[1], Philipp Kron[1]

[1]*Klinik für Allgemeine, Viszeral- und Transplantationschirurgie, Universitätsklinikum Tübingen, Tübingen, Deutschland*, [2]*Institut für Anästhesiologie, Universitätsspital Zürich, Zürich, Schweiz*

**Background:**
Entrustable Professional Activities (EPAs) are becoming increasingly popular as a framework for clinical assessment and are seen as a valuable tool in advancing competency-based medical education (CBME). Many surgical residency programs worldwide have adopted EPAs to evaluate and provide feedback on their residents' performance. This structured, competency-based approach allows learners to build and refine their knowledge and skills under supervision. Despite the global implementation and benefits of EPAs, their usage in German surgical centers remains limited. The primary goal of this study is to assess the extent of EPA usage in German hospitals. In the second phase, the study seeks to identify the needs and expectations of stakeholders regarding a more structured surgical curriculum. In the final phase, the study aimed to implement EPAs using the "Prepared App" at the Department of General, Visceral and Transplant Surgery at Tübingen University Hospital.

**Materials and methods:**
A survey was conducted to evaluate the application of EPAs in major centers across Germany. A structured questionnaire was developed to identify barriers and facilitators for successful and sustainable EPA implementation. Following this, EPAs were introduced using the "Prepared App" at the Department of General, Visceral and Transplant Surgery at Tübingen University Hospital.

**Results:**
This manuscript outlines the current landscape of EPA adoption in German surgical centers. EPAs represent a significant change in the evaluation of surgical residents. The survey findings clearly demonstrate strong support for EPAs, highlighting their value in providing structured feedback and improving the surgical training curriculum.

**Conclusion:**
While the advantages of digital EPAs are evident, their integration into surgical core training in Germany remains in its early stages. However, broader implementation will foster acceptance and help streamlining surgical education. Overall, EPAs signal a major shift towards competency-based training and have the potential to enhance surgical residency programs by providing structured, objective feedback to residents.

**Lessons from the Implementation of RAMIE in a Single Tertiary Referral Centre – a Risk-Adjusted Learning Curve**
ID: 893

**Kategorie: DGCH - Herausforderungen in der chirurgischen Weiterbildung**

Philippa Seika[1], Max Magnus Maurer[1], Ramin Raul Ossami Saidy[1], Paul Ritschl[1], Jonas Raakow[1], Johann Pratschke[1], Christian Denecke[1]

[1]*Charité - Universitätsmedizin, Chirurgische Klinik, Berlin*

**Background:**
Robot-assisted minimally invasive esophagectomy (RAMIE) is an evolving technique in the management of oesophageal cancer, with a distinct learning curve. This study aims to assess the learning curve associated with RAMIE and its impact on patient outcomes.

**Materials and methods:**
A retrospective analysis was conducted on 84 patients who underwent RAMIE between 2018 and 2024. Patient demographics, tumour characteristics, and surgical parameters were evaluated. The RA-CUSUM methodology was used to monitor patient outcomes, and varying-coefficient logistic regression models were applied to establish the learning curve for RAMIE proficiency.

**Results:**
Technical competence, in terms of operative time, was achieved after 32 cases. The learning curve for major complications was completed after 38 consecutive cases. The incidence of textbook outcome (TO) increased significantly after 42 cases, with TO rates improving from 28.00% in the initial phase to 50.00% in the later phase (p=0.039). Anastomotic leakage rates remained stable across the learning curve (9.25%), with no significant difference in leakage between early and late cases (p=0.648). Pulmonary complications constituted most postoperative issues, but their incidence decreased after reaching competence. After a median follow-up of 18 months, no significant differences in overall survival (p=0.489) or disease-free survival (p=0.387) were observed between the early and late phases of the learning curve.

**Conclusion:**
The learning curve for RAMIE, in terms of operative time and major complications, was completed after 32 to 38 cases. Implementation of RAMIE has a considerably shorter LC as compared to MIE. The achievement of TO required more extensive experience, with competence reached after 42 cases. These findings suggest that RAMIE can be implemented in medium to high volume centres while maintaining patient safety and oncological outcomes.

### Prediction of survival in colorectal cancer patients on the basis of body mass index and lymph node yield – a retrospective single center analysis over a quarter of a century

ID: 6

**Kategorie: DGCH - Langzeitergebnisse Checklisten in der Chirurgie**

Tamara Braunschmid[1], Stefan Stättner[2], Alexandra Graf[3], Jagdeep Bhangu[1], Thomas Grünberger[4], Daniela Kandioler[5]

[1]*Klinik Floridsdorf - Wiener Gesundheitsverbund, Chirurgie, Wien, Österreich*, [2]*Salzkammergut Klinikum Vöcklabruck, Vöcklabruck, Österreich*, [3]*Medizinische Universität Wien, Statistik, Wien, Österreich*, [4]*Klinik Favoriten - Wiener Gesundheitsverbund, ehemaliges Kaiser Franz Josef Spital, Chirurgie, Wien, Österreich*, [5]*Medizinische Universität Wien, Chirurgie, Wien, Österreich*

**Background:**

Colorectal Cancer (CRC) is one of the most frequent cancer entities in the western world. Obesity is one of the major risk factors for CRC. In contrast, mild obesity seems to have a positive effect on postoperative outcome, which is known as the “obesity paradox”. Also, the number of resected lymph nodes (LN) during surgery is very important for correct staging. The aim of the present project is to evaluate the impact of BMI and LN-yield on long term overall survival (OS) of CRC patients treated with curative intent.

**Materials and methods:**

Patients treated for CRC between 1998 and 2011 at the Klinik Favoriten in Vienna, Austria were analyzed in this retrospective single-center cohort analysis concerning OS.

**Results:**

A total of 1,192 patients (47.7% female) were included. Median age was 70.3 years, median BMI was 25.7, median number of LN examined was 22, median survival was 76 months, median follow up was 11.3 years. Underweight patients had the highest, overweight patients the lowest risk of death and the shortest postoperative hospital stay. In patients with a smaller LN-yield or a higher number of positive LN an increase of mortality was found. There was no difference in number of harvested LN within age groups or BMI categories.

**Conclusion:**

The present study shows the analysis of 1,192 CRC patients with a median follow-up of 11.3 years (range: 0-23.2 years) which is – to the author’s knowledge – the longest FU period of a single center cohort with more than 1,000 CRC patients ever published. BMI has a considerable influence on OS in certain subgroups of CRC patients. Underweight patients suffering from colorectal cancer have a significantly higher mortality whereas overweight patients have the lowest risk to die compared to other BMI groups. Especially in colon cancer patients BMI showed a significant association with OS with no gender specific differences. With these findings the present study supports the theory of the “obesity paradox” by adding evidence to that topic with over 1,000 patients analyzed and followed for a quarter of a century. Furthermore, patients with colon carcinoma have a higher mortality than patients with rectal carcinoma. Also, colorectal cancer patients with a smaller lymph node yield or more positive lymph nodes have an unfavorable prognosis.

**Single-center study of the current communication patterns in the operating theatre.**
ID: 810

**Kategorie: DGCH - Langzeitergebnisse Checklisten in der Chirurgie**

Alexander Bungert[1], Jan Philipp Ramspott[1], Alina Knipping[1], Benjamin Struecker[1], Andreas Pascher[1], Jens Peter Hölzen[1]

[1]*Department of General, Visceral and Transplant Surgery, University Hospital Muenster, Muenster, Deutschland*

**Background:**
Communication in hospitals or in the operating theatre has a significant impact on patient safety in surgery. As the operating theatre is one of the high-risk areas in the hospital, many tools were introduced to ameliorate communication and to avoid communication associated failures jeopardizing patients' health. Despite introduction of the well-known World Health Organization (WHO) Surgical Safety Checklist (SSC) or the Crisis Resource Management (CRM) communication seem to be improvable.

**Materials and methods:**
In this study, we analysed the status quo of the current employee satisfaction regarding communication, atmosphere and implementation of the SSC in the operating theatre at a university hospital in Germany. We also asked to what extent the employees were known by name and whether an improvement could be expected by identifying respective persons with name tags. A questionnaire of 19 questions was sent to 611 employees of all surgical disciplines including surgeons, nurses, service and cleaning staff. Time limit was set to 4 weeks after the first email was sent, two reminder emails were planned. Time to fill out the questions was estimated to be 7 minutes with the possibility of free text for further suggestions made by the interviewees. No financial incentive was made.

**Results:**
Initial results show that, although implementation of the SSC was mainly carried out, the mutual introduction by name did not always occur even when the employees did not know each other before. This led to dissatisfaction and misguided communication. However, name tagging in the operating theatre was considered as one tool to ensure mutual familiarity by name.

**Conclusion:**
Communication in the operating theatre is still one human factor with great impact on patients' safety and health. Current strategies to overcome error-prone inter- and intraprofessional communication are urgently needed. Name tagging could represent one tool to improve interaction promptly without significant effort.

### Estimation of optimal Surgical Minimum Caseloads for major oncological resections using a Population Attributable Fraction Model

ID: 69

**Kategorie: DGCH - Mindestmengen unter den Vorzeichen der Einführung der Leistungsgruppen**

Konstantin Lukas Uttinger[1], Mohammad Ali Mansournia[2], Armin Wiegering[1]

[1]*Uniklinik Frankfurt, Klinik für Allgemein-, Viszeral- und Transplantationschirurgie, Frankfurt am Main, Deutschland*, [2]*Department of Epidemiology and Biostatistics, Teheran, Iran*

**Background:**
The introduction of surgical minimum caseloads (SMC) has been an effective tool in centralization programs with the overall aim of optimizing patient outcome. However, there is no evidence-based approach to determine optimal threshold SMC are optimal with regards to in-hospital mortality.

**Materials and methods:**
A statistical modelling based on anonymized hospital billing data (2011-2022) was used to estimate optimal SMC with regards to in-hospital mortality in pulmonary, esophageal, gastric, pancreatic, colon, and rectal resections. Patient record identification was done using German Cancer Society certification criteria. Current SMC per year were 20 in esophageal, gastric, pancreatic and rectal, 30 in Colon, and 75 in pulmonary resections. Non-fulfillment of SMC of 5 to 100 resections per year was defined as risk factor in a population attributable fraction (PAF) model. Based on this PAF model, an optimal SMC was obtained using a linear trend approach in a training cohort (2011-2018). The primary endpoint was PAF of non-fulfillment of the current SMC in the training cohort and PAF of an optimal SMC in a validation cohort (2019-2022). Secondary endpoints were resulting attributable deaths and the difference between the driving distance to the treating hospital and to the closest hospital fulfilling current and optimal SMC.

**Results:**
A total of 824,535 patient records were analysed. The number of hospitals performing at least one resection per year between 2011-2018 was lowest for pulmonary resections (median 328 hospitals, min 321, max 334) and highest for colon resections (median 985 hospitals, min 928, max 1,047). Overall in-hospital mortality ranged between 2.9% (2,736 of 94,417) in pulmonary resections and 7.3% (5,734 of 78,206) in pancreatic resections. A multivariable regression model for in-hospital mortality was built adjusting for age, sex, resection extent, comorbidity, and main diagnosis (esophageal and pancreatic resections), resulting in Risk Ratio (RR) of in-hospital mortality for non-fulfillment of the current SMC and, consequently, PAF of SMC non-fulfillment, which ranged between 7.9% (95% Confidence Interval, CI, 2.1%-13.7%) in Colon resections, equal to 617 (95% CI 164-1,070) attributable deaths, and 23.1% (95% CI 12.7%-33.4%) in gastric resections, equal to 687 (95% CI 378-662) attributable deaths in the training cohort.
According to the linear trend approach, optimal SMC were 50 per year in pulmonary resections, 31 per year in esophageal resections, 31 per year in gastric resections, 48 per year in pancreatic resections, 28 in colon resections, and 43 per year in rectal resections. The PAF of non-fulfillment of the optimal SMC was lowest in colon resections (8.8%, 95% CI 1.0%-16.5%) and highest in pancreatic resections (30.6%, 95% CI 22.8%-38.5%). Attributable deaths were lowest (147, 95% CI 22-272) in pulmonary resections and highest in pancreatic resections (798, 95% CI 593-1,004). In the validation cohort, the potential median difference in the driving distance (patient to treating hospital vs patient to optimal SMC fulfilling hospital) ranged between -3.5km (IQR -16.2 km- +0.2 km) in colon resections and +39.1 km (IQR +0.3 km- +89.5 km) in rectal resections.

**Conclusion:**
On the basis of this analysis with its restrictions due to the nature of retrospective data, this work presents optimized minimum caseload thresholds in major surgery with regards to in-hospital mortality, and analyses its practicability with regards to driving distance. Beyond this, this work introduces an approach for optimal SMC determination in procedures with a volume-outcome relationship in general.

**Impact of sleep deprivation on laparoscopic performance: a prospective, randomized crossover trial**
ID: 255

**Kategorie: DGCH - Minimalinvasive, computer- und Telematik-assistierte Chirurgie**

Jean-Paul Bereuter[1], Alexa Fries[1], Isabell Grummt[1], Mark Enrik Geissler[1], Rona Geissler[1], Sofia Schmidt[1], Nathalie Buck[1], Juliane Weiß[1], Grit Krause-Jüttler[1], Jürgen Weitz[1 2], Marius Distler[1 2], Florian Oehme[1], Felix von Bechtolsheim[1 2]

[1]*Department of Visceral, Thoracic and Vascular Surgery, Faculty of Medicine and University Hospital Carl Gustav Carus, Technische Universität Dresden, Dresden, Deutschland*, [2]*Centre for Tactile Internet with Human-in-the-Loop (CeTI), Technische Universität Dresden, Dresden, Dresden, Deutschland*

**Background:**
Laparoscopic surgery is a commonly used technique that requires several skills including manual dexterity, precise hand-eye coordination, and cognitive focus. Any stressor that affects these essential skills can potentially compromise the safety and efficacy of laparoscopic procedures. During night shifts, surgeons experience prolonged periods without sleep, which might impact these skills and therefore also their laparoscopic performance. However, to date there is limited objective data investigating this issue. Therefore, the aim of this study was to elucidate the effect of sleep deprivation on laparoscopic surgical performance based on tissue handling parameters.

**Materials and methods:**
A total of 55 medical students and 20 abdominal surgeons of the medical faculty in Dresden participated in this single-center, prospective, randomized cross-over trial. All students were laparoscopic novices, who participated in a structured laparoscopic training curriculum until reaching a predefined proficiency. For the trial, all participants had to perform three laparoscopic tasks (Peg transfer, circle cutting and laparoscopic suture) once after a night without sleep (sleep deprived measurement) and once without any sleep deprivation (control measurement). During both the intervention and the control measurement, all participants performed the three laparoscopic tasks on the ForceSense system (Medishield B.V., Delft, The Netherlands). The ForceSense system was used to measure the force exertion (peak and mean non-zero-force) as well as motion parameters (path length, mean instrument speed, volume of motion, etc.). Primary endpoints were tissue handling defined by force exertion, instrument motion, task time and error rate. The sleep impairment was assessed by biological parameters like heart rate and blood pressure and psychological questionnaires such as the Stanford Sleepiness Scale.

**Results:**
All participants completed the trial and were subsequently assessed. To evaluate the effects of sleep deprivation, both biological and psychological parameters were measured. During the sleep deprivation phase, novices exhibited a significantly lower heart rate (HR) compared to the control measurement (control: 81.8 bpm vs. sleep-deprived: 76.4 bpm, $p = 0.017$). In contrast, no significant difference in heart rate was observed among surgeons (control: 67.8 bpm vs. sleep-deprived: 69.8 bpm, $p = 0.48$). When examining subjective sleepiness, both novices (control: 2.82 vs. sleep-deprived: 3.31, $p < 0.001$) and surgeons (control: 2.4 vs. sleep-deprived: 2.82, $p = 0.004$) reported significantly higher levels of sleepiness during the sleep deprivation phase. To assess how this increased sleepiness impacted the participants' laparoscopic skills, primary endpoints were analyzed. Overall, only isolated significant differences were noted. For example, during the sleep deprivation phase, novices demonstrated a significantly shorter instrument path length in the Peg transfer task compared to the control measurement (control: 4229 mm vs. sleep-deprived: 3906 mm, $p = 0.01$). Additionally, the task completion time for the circle cutting task was significantly shorter in the sleep-deprived condition (control: 205.5 s vs. sleep-deprived: 188.2 s, $p = 0.02$). Furthermore, the novices' mean non-zero force during the suture and knot tying task was significantly lower under sleep deprivation (control: 0.84 N vs. sleep-deprived: 0.75 N, $p < 0.001$). Other force and motion parameters, as well as the error rate, were unaffected by sleep deprivation in surgical novices. Interestingly, surgeons exhibited similar isolated differences to those observed in novices.

**Conclusion:**
Our study revealed neither adverse nor beneficial systemic effects of sleep deprivation on laparoscopic surgical skills. In general, based on our results, laparoscopic motion and tissue handling skills of surgical novices do not seem to deteriorate significantly under the impact of sleep deprivation. However, even when not generally harming the students' and surgeons' tissue handling skills, the observed spot wise effects warrant attention.

### Studying the Hepatic Resection Angle in Laparoscopic Partial Hepatectomy

ID: 401

**Kategorie: DGCH - Minimalinvasive, computer- und Telematik-assistierte Chirurgie**

Hiroyasu Nishimaki[1], Keisuke Fukushima[1], Rikiya Kanba[1]

[1]*Osaki City Hospital, Furukawa, Japan*

**Background:**

In partial hepatectomy, tumor resection without excess or deficiency is more important than in systematic hepatectomy. Therefore, determining the appropriate resection line and securing the resection margin are important for a safe surgery. In recent years, partial hepatectomy has often been performed laparoscopically. In laparoscopic surgery, determining the appropriate resection margin for hepatectomy is more important because of the limited movement of the laparoscopic forceps. Therefore, we aimed to determine the optimal resection margin for liver resection.

**Materials and methods:**

We selected 16 cases of laparoscopic partial hepatectomy for a single tumor at the first time from 59 cases of hepatectomy performed at our hospital from 2021 to 2023. We also determined the maximal diameter whole-tumor section from the resected specimen photographs retrospectively. We set the two liver surface ends at points A and B; the point of the maximal diameter from the liver surface to the dissection surface was point C. The three points formed a triangle and the internal angle (dissection angle) of point C was measured. The data was divided into two groups according to the divided angle 90°, 100°, 110°, and 120°. We determined the better angles. We examined patients' background, such as age, gender, and body-mass indices, and short-term outcomes, such as postoperative complications and length of hospital stay. We also examined the operation time and intraoperative bleeding as a surgical result. As the dissection surface length changed depending on the tumor diameter, we divided the operation time and bleeding volume by the tumor diameter to obtain the modified operation time and intraoperative bleeding.

**Results:**

Patient backgrounds showed no differences between the four studies. Although the operation time was significantly reduced in the 90° groups ($p = 0.04$), the intraoperative bleeding showed no significant differences, although the a tendency for less bleeding was observed ($p = 0.08$). No differences were observed in the other groups. Short-term outcomes such as postoperative complications and length of stay in hospital showed no differences in all groups.

**Conclusion:**

A reduced operation time was observed in the 90° group. According to the Japanese classification of the liver tumor, the maximal diameter whole-tumor section is considered to be the computed tomography cross-section. Therefore, measuring resection distance from a preoperative computed tomography scan and determining the resection line is suggested, such that the separation angle is 90°. Hence, minimally invasive surgeries can be performed without excess or deficiency.

**Oncological survival in pancreatic head ductal carcinoma: hybrid minimally invasive versus open pancreatoduodenectomy - a single centre analysis**
ID: 736

**Kategorie: DGCH - Minimalinvasive, computer- und Telematik-assistierte Chirurgie**

Johannes Kaiser[1], Simon Kuesters[2], Stefan Fichtner-Feigl[1], Esther Anna Biesel[1], Sophia Chikhladze[1], Uwe A. Wittel[1]

[1]*Universitätsklinikum Freiburg, Klinik für Allgemein- und Viszeralchirurgie, Freiburg, Deutschland*,
[2]*RKH Fürst-Stirum-Klinik Bruchsal, Klinik für Allgemein- und Viszeralchirurgie, Bruchsal, Deutschland*

**Background:**
Surgery remains the only curative treatment option for pancreatic head cancer. Laparoscopic surgical techniques are increasingly used, in line with the development of visceral surgery as a whole. The success of surgery is measured by the oncological outcome. Aim of this study is to evaluate the oncological outcome and survival after laparoscopic assisted resection of the pancreatic head compared to open surgery for ductal adenocarcinoma.

**Materials and methods:**
Data were collected in a prospectively maintained database. Perioperative and oncological outcomes of 182 laparoscopic pancreatic head resections for ductal carcinoma were compared with 585 open pancreatic head resections. The laparoscopic procedures were performed between 2010 and 2022, the open procedures between 2002 and 2022.

**Results:**
Laparoscopic procedure was significantly superior in terms of intraoperative blood loss (575 vs. 600 ml, $p = 0.021$) and operative time (413 vs. 427 min, $p = 0.033$). Tumour size (25 vs. 27 mm, $p = 0.028$), need for portal vein resection ($p = 0.009$) and blood transfusion ($p = 0.004$) were significantly greater in the open group ($p = 0.009$). The resection margin remained negative significantly more often in the laparoscopic group (159 [87%] vs. 449 [77%], $p < 0.001$). There was no difference in postoperative mortality and morbidity. Postoperative survival at 5 years was significantly better in the laparoscopic group (37% vs 15%, $p < 0.001$).

**Conclusion:**
In our cohort, patients who underwent hybrid laparoscopic resection of pancreatic head cancer showed a significantly improved oncological outcome. Most of these effects are due to selection bias, which is not captured by the clinical parameters used to date. Our results highlight the need for additional prognostic factors in pancreatic cancer.

**Beyond Traditional Repair: Assessing eTEP and Sublay Techniques for Ventral Hernias**
ID: 793

**Kategorie: DGCH - Minimalinvasive, computer- und Telematik-assistierte Chirurgie**

Phillip Looft[1], Fadl Alfarawan[1], Nader El-Sourani[2]

[1]*Klinikum Oldenburg AöR, Oldenburg, Deutschland*, [2]*Universitätsklinikum Münster, Münster, Deutschland*

**Background:**
Managing ventral hernias, which significantly affect the anterior abdominal wall, requires effective surgical intervention to alleviate discomfort and prevent complications. This study compares two hernia repair techniques: the minimally invasive Enhanced-view Totally Extraperitoneal (eTEP) approach and the Sublay method, known for durable repairs through mesh integration. Despite their advantages, consensus on their efficacy, complication rates, and impact on quality of life is lacking. Our research systematically compares eTEP and Sublay techniques, evaluating early clinical outcomes, recovery times, and patient satisfaction to provide evidence-based recommendations for optimal surgical strategies, aiming to enhance patient outcomes and inform global surgical protocols.

**Materials and methods:**
A retrospective review was conducted to compare patients who underwent eTEP versus sublay hernia repair between July 2019 and March of 2023 at the surgical department of Klinikum Oldenburg. Patient demographics and perioperative data were collected and analyzed using univariate analysis.

**Results:**
A total of 139 patients were analyzed, with 92 undergoing eTEP repair and 47 undergoing sublay repair. Both groups were comparable in demographic and clinical characteristics, with no significant differences in age (p=0.207) or BMI (p=0.277).
Significant differences were found in defect size (median 6 cm² for eTEP vs. 16 cm² for Sublay, p<0.028) and mesh size (median 450 cm² for eTEP vs. 150 cm² for Sublay, p<0.001), indicating different reinforcement approaches. Operation time was similar (p=0.119), and postoperative pain levels over three days showed no significant differences (VAS D1 p=0.884; VAS D3 p=0.649).
Intraoperative complications were 2.17% for eTEP and 6.38% for Sublay (p=0.207, not significant). Postoperative complications were significantly lower in the eTEP group (7.61% vs. 23.4%, p=0.009).
Hospital stay was shorter for eTEP patients (median 3 days vs. 5 days, p<0.001), reflecting potentially faster recovery and fewer complications.

**Conclusion:**
eTEP is a safe and effective option for ventral hernia repair. It is associated with a shorter postoperative hospital stay and potentially lower complication rates compared to the sublay technique. Although eTEP has a marginally longer operation time, its benefits in terms of recovery and reduced complications make it a favorable choice for many patients.

**Hybrid treatment of intrathoracic subclavian artery aneurysms**
ID: 877

**Kategorie: DGCH - Minimalinvasive, computer- und Telematik-assistierte Chirurgie**

Peter Donndorf[1 1], Clemens Schafmayer[1], Justus Groß[1]

[1]*Universitätsmedizin Rostock, Klinik für Allgemein-, Viszeral-, Thorax-, Gefäß- und Transplantationschirurgie, Rostock, Deutschland*

**Background:**
Intrathoracic subclavian artery (SAA) aneurysms are rare clinical findings. However due to their life- and limb threatening nature invasive treatment is indicated for large aneurysms. As open surgical procedures are associated with a relevant surgical trauma and endovascular attempts addressing only the deseased subclavian artery are frequently uncertain without adequate landing zones in large aneurysms hybrid approaches combing TEVAR and surgical debranching offer a reasonable therapeutic alternative in the era of endovascular aortic repair.

**Materials and methods:**
We present here our experience with hybrid treatment of intrathoracic SAAs by demonstrating two cases.

Case 1: A 63year old female was referred to our department with a pulsatile left supra-clavicular tumor for further diagnostic and treatment.
Medical history included Marfan syndrome and replacement of the aortic valve and the ascending aorta well as open thoracoabdominal aortic replacement. Contrast enhanced Computed Tomography (CT) revealed a giant aneurysm of the proximal and middle left subclavian artery. Due to extensive previous intrathoracic surgery direct open surgical repair was discarded and we opted for a hybrid approach combining a thoracic aortic stent graft covering the aortic origin of the left subclavian artery and left carotid-subclavian bypass as well as transposition of the left vertebral artery into the left common carotid artery for SAA exclusion.
Case 2: A 61year old male was diagnosed with an asymptomatic but large (32mm) intrathoracic aneurysm of an aberrant right subclavian artery (Kommerell's diverticulum) during diagnostic work-up prior surgery due to a symptomatic carotid artery stenosis.
Following surgical treatment of the carotid artery the SAA was treated with a combination of right carotid-subclavian artery bypass, plug-embolization of the distal intrathoracic right subclavian artery and proximal coverage of the aberrant right subclavian utilizing a thoracic aortic stent graft.

**Results:**
Both patients had an uneventful postoperative course and were discharged at post-operative day six. Control CT prior hospital discharge showed sufficient exclusion of the subclavian aneurysm and unobstructed perfusion of the bypassed/ transposed head and arm vessels.

**Conclusion:**
Large intrathoracic subclavian artery aneurysms are rare but can occur in certain patient subgroups, e.g. patients with Marfan syndrome or arch abnormalities. Our integrated concept combines well established endovascular (TEVAR) and open (supra-aortic debranching) procedures in order to achieve safe and reliable aneurysm exclusion. Although endovascular procedures should always be considered carefully for example when treating aneurysmal disease in Marfan patients, we believe that in cases like ours with a high technical risk of complete open surgical repair the use of a routine endovascular procedure like TEVAR in combination with a tailored open surgical part for a less invasive and still efficient aneurysm treatment is justified. Our cases are good examples how combining endovascular and open surgical procedures facilitates safe and efficient treatment even of rare and anatomically challenging vascular pathologies.

### Thoracoscopic repair of recurrent CDH is associated with a significantly lower complication rate and shorter ICU and hospital stay: A prospective, propensity score-matched analysis

ID: 918

**Kategorie: DGCH - Minimalinvasive, computer- und Telematik-assistierte Chirurgie**

Christel Weiss[1], Christoph Mohr[2], Richard Martel[2], Michaela Klinke-Petrowsky[2], Jin Rhee[2], Kathrin Zahn[2], Thomas Schaible[3], Michael Boettcher[2], Lydia Beck[2], Julia Elrod[2]

[1]*Statistik und Biomathematik , Mannheim, Deutschland*, [2]*Kinderchirurgie, Mannheim, Deutschland*, [3]*Neonatologie, Mannheim, Deutschland*

**Background:**
Congenital diaphragmatic hernia (CDH) is a severe neonatal malformation associated with high mortality and morbidity. A common long-term complication is recurrence after surgical repair. Similar to the primary operation, recurrence can be addressed through either open or minimally invasive surgery. We hypothesized that a thoracoscopic minimally invasive approach (MITS) could lead to faster recovery and fewer complications, so we implemented it as the primary approach for recurrent CDH repair at our center. Since this is a novel technique, we aimed to compare the postoperative outcomes of both techniques.

**Materials and methods:**
A prospective, propensity score-matched analysis was conducted on pediatric patients who underwent recurrent CDH repair at the University Hospital Mannheim, University Heidelberg, between 2013 and 2023, to compare the outcomes of laparotomy vs. MITS Patients were categorized based on the surgical technique employed. Comparative analysis, including propensity scoring, encompassed outcome measures such as duration of ICU and hospital stays, rate of complications, and operative duration.

**Results:**
In total, 703 patients were treated for CDH; of whom 69 children underwent laparotomy (56) or MITS (16) for CDH recurrence. After propensity score matching, the MITS group demonstrated a significantly shorter duration of surgery (183.8±68.5 vs. 243.2±88 min, p= 0.042), reduced ICU stay (1.6 ±3.2 vs. 7.5±16.3 days, p = 0.011), and decreased overall hospital stay (8.4±8.0 vs. 15.5±8.8 days, p = 0.001). Moreover, the postoperative complications were significantly lower in the MITS group (21.43% vs. 73.68%, p = 0.003). In the observation period

**Conclusion:**
Preliminary findings suggest that MITS for recurrent CDH is a safe and beneficial alternative. It is associated with shorter operation times, reduced ICU and overall hospital stays, and fewer complications compared to laparotomy. However, as the sample size and observation period are limited, these findings require further confirmation through additional studies to validate the long-term safety and efficacy of this approach.

### Robotic assisted repair of a right sided Pantaloon, femoral and obturator hernia
ID: 929

**Kategorie: DGCH - Minimalinvasive, computer- und Telematik-assistierte Chirurgie**

Hugo Bonatti[1], John Kessler[1], Stephen Kavic[1]

[1]*University of Maryland School of Medicine, Surgery, Baltimore, Vereinigte Staaten*

**Background:**
Only few cases of combined inguinal and femoral or obturator hernias have been reported. Laparoscopic and robotic repair seems safe and feasible in these cases.

**Materials and methods:**
A 74 year old male presented with significant pain and a bulge protruding in the right groin reaching into the scrotum.

**Results:**
He underwent robotic assisted inguinal hernia repair. On preperitoneal dissection a right pantaloon and in addition, a femoral hernia was found. Whilst developing the medial channel to below the pubic bone, an obturator hernia was found. A large self fixating mesh was placed covering all four hernia defects with appropriate overlap in all directions. The patient was discharged within 3 hours after surgery and had an uneventful recovery.

**Conclusion:**
To the best of our knowledge this is the first “quadruple” groin hernia repair using the robotic approach.

### Monocentric prospective randomized study to assess the impact of the on-screen use of a virtual 3-dimensional liver model on spatial perception during laparoscopic surgery on a liver phantom

ID: 984

**Kategorie: DGCH - Minimalinvasive, computer- und Telematik-assistierte Chirurgie**

Anders Grabenkamp[1], Roger Wahba[2], Rabi Datta[1]

[1]*Uniklinik Köln, AViszeralchirurgie, Köln, Deutschland*, [2]*Helios Klinikum Berlin-Buch, Berlin, Deutschland*

**Background:**
Minimally invasive surgery is of great importance in modern abdominal surgery and the number of laparoscopic liver surgeries increases steadily. Nevertheless, there are still disadvantages such as loss of perception and orientation during laparoscopic procedures. Finding ways to compensate for those factors are part of current research. Furthermore, the learning curve regarding laparoscopic liver surgery is very steep, especially for novices. Therefore, this study aims to show benefit for the use of a virtual on-screen 3D liver model while performing a laparoscopic liver procedure.

**Materials and methods:**
A randomized cross-over, single-institution, single-blinded trial is designed. The aim of the study is to compare the performance of localizing liver lesions ultrasound guided by using a virtual on-screen 3D liver model in combination with CT scan data vs. by only using the CT scan. Primary outcome parameters are the time needed to find the lesions and correctness of finding the searched lesions. It compares primary outcome performance measured as time and errors using an on-screen virtual 3D liver model to localize lesions ultrasound guided on a liver phantom in a box trainer, compared to the use of only CT scan data. Secondary outcome parameters are cognitive workload measured by NASA Task Load index and usability measured by the System Usability Score. Subjects will be randomized into two groups, one will start by only using the CT scan data “CT first”, the other by both using the CT scan and the virtual 3D model “3D model first”. Second run will be vice versa. Subjects will be randomized to start with either “CT first” or “3D model first”. They should name the segment of a shown target lesion and present a resection plan. Then they localize the lesion in the liver phantom ultrasound guided with the help of the available imaging as described before. After localizing the lesions, cognitive workload and usability will be measured. Assuming a correlation of 0.5 between measurements per subject, a sample size of 36 per stratum is required to detect a standardized effect of 0.5 (including an additional 5% for non-parametric approach with a power of 80% at a two-sided type I error of 5%, 72 subjects need to be enrolled. Final data analyses will be performed pseudonymized and blinded.

**Results:**
At the time of this Abstract Submission, full data analysis is yet to finish. Preliminary data show a high significance for the use of the virtual on-screen 3D liver model being superior to the use of CT data only for finding searched liver lesions correctly. Moreover, medical students/surgical novices profit from using the 3D model even more than trained liver surgeons.

**Conclusion:**
Laparoscopic liver surgery is becoming increasingly important due to its advantages for patients like reduced intraoperative blood loss and shortened hospital stay. For surgeons, laparoscopic surgery is known to be challenging especially for less experienced surgeons/novices because of the lack of spatial perception and orientation. Apart from modern display systems, the use of technical innovations such as virtual models are promising to provide guidance during the laparoscopic surgery.
This study evaluates the impact regarding performance and workload of an on-screen use of a 3D virtual liver model during laparoscopic surgery on a liver phantom. The hypothesis is that the use of the virtual 3D model improves intraoperative orientation which should result in a better performance and reduction of workload.
If the benefits of using the virtual model can be proven it is conceivable to conduct further clinical studies or engage in further development of the virtual model.
The authors hope to generate evidence supporting the benefits of using an intraoperative 3D model during laparoscopic liver surgery, especially for novices. This work aims to pave the way for future clinical trials to simplify the learning process of laparoscopy and, ultimately, improve patient safety.
Trial Registration: The study is registered at ClinicalTrials.gov (trial number NCT05389241).

In-/Exclusion Criteria
Demographic Questionnaire
Training
Randomization
CT only
CT and 3D model
Group CT first (n=36)
Anatomical structure
Resection plan
Target Lesions A+B on liver phantom
Group 3D model first (n=36)
Anatomical structure
Resection plan
Target Lesions A+B on liver phantom
Modification of Resection plan?
NASA Task Load Index & System Usability Scale
Resection plan
Target Lesions C+D on liver phantom
Resection plan
Target Lesions C+D on liver phantom
Modification of Resection plan?
NASA Task Load Index & System Usability Scale
pseudonymized statistical evaluation

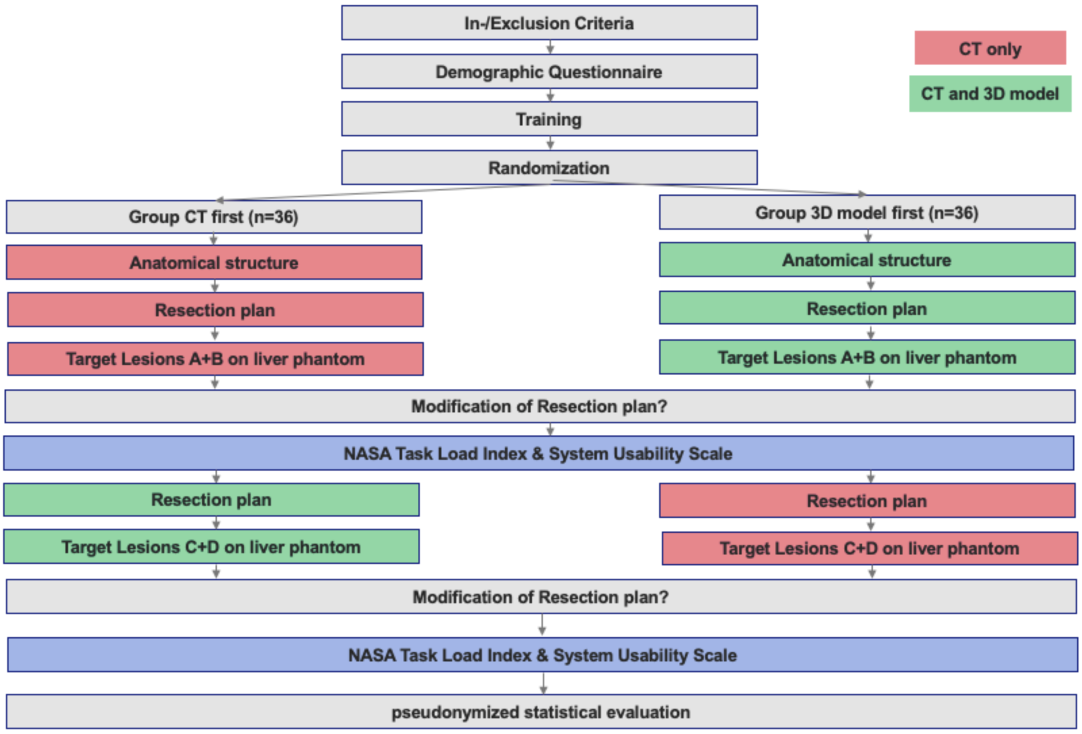

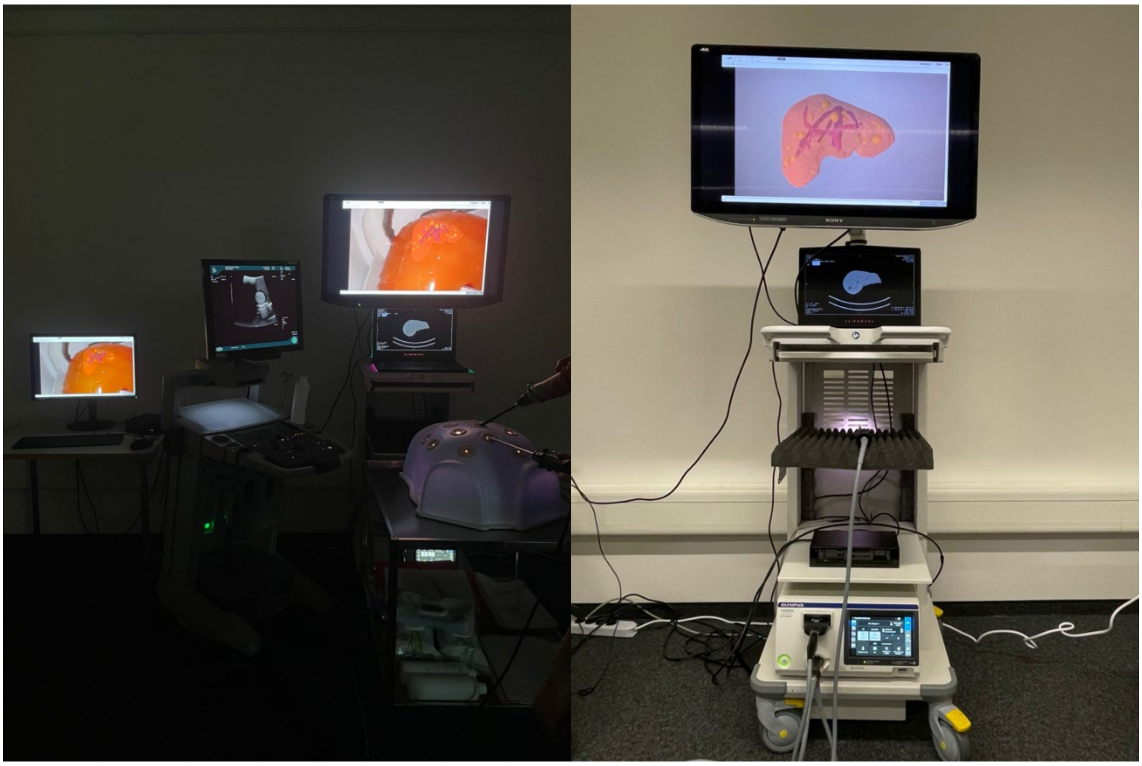

### 2 year results of an interprofessional training ward (ITW) at the University Hospital Münster
ID: 263

**Kategorie: DGCH - Moderne Konzepte zur Nachwuchsgewinnung**

Nader El-Sourani[1], Christian Hellmann[1], Angelika Maase[1], Madeleine Barthmuß[1], Andreas Pascher[1]

[1]*Universitätsklinikum Münster, Klinik für Allgemein-, Viszeral - und Transplantationschirurgie, Münster, Deutschland*

**Background:**
Interprofessional education (IPE) is essential in medical training to enhance collaborative skills among healthcare professionals. An Interprofessional Training Ward (ITW) was established at the Department for General -, Visceral - and Transplantation Surgery, University Hospital Münster to promote practical learning and collaboration between medical and nursing students, aiming to improve clinical competence and foster a positive learning atmosphere.

**Materials and methods:**
The ITW was staffed by two medical and two nursing students, who worked together under the supervision of senior healthcare professionals. Every rotation lasted for 4 weeks with participants taking care of a total of 6 patients. A pre-post design was employed to evaluate the ITW's effectiveness. Data were collected using a standardized questionnaire based on a Likert scale from 1 (strongly disagree) to 5 (strongly agree). The questionnaire assessed subjective learning experiences (7 items) and learning atmosphere (7 items) before and after participation in the ITW. Feedback was gathered at the beginning and end of each rotation from February 2022 to December 2023.

**Results:**
A total of 80 medical and nursing students participated in this study. The implementation of the ITW led to significant improvements in both subjective learning experience and learning atmosphere. The average score for all items assessing subjective learning experience increased from 3.18 to 4.29, with "taking on responsibility" being the best scored item rising from 2.83 to 4.38. The average score for all items assessing learning atmosphere improved from 3.14 to 3.93, with "interprofessional collaboration" being the best scored item increasing from 2.96 to 4.26.

**Conclusion:**
The ITW effectively improved subjective learning experience and learning atmosphere, especially the items interprofessional collaboration and taking on responsibility, underscoring the importance of interprofessional settings in preparing future healthcare professionals for collaborative clinical practice.

### Inspiring and maintaining passion for surgical specialities: a student-led approach to encourage surgical careers

ID: 279

**Kategorie: DGCH - Moderne Konzepte zur Nachwuchsgewinnung**

Kira Meißner[1], Hajera Khaleqi[1 2], Pauline Schuppert[1 2 3], Lucie Rohwäder[1 2], Merle Juschka[1], Saman Keshtkaran[1], Abdulrahman Al-Harazi[1], Antonia Reers[1], Lara Cierna[1], Gaby Lee[1], Kim Seifert[1], Amila Cizmic[4], Anas Amin Preukschas[4], Felix Nickel[4], Andreas Lindner[5], Johanna Hagens[2], Melanie Le[2], Christian Tomuschat[2], Hans Christian Schmidt[1 2]

[1]*SimLab – Students for Surgery, University Medical Center Hamburg-Eppendorf, Hamburg, Deutschland*, [2]*Department of Pediatric Surgery, University Medical Center Hamburg-Eppendorf, Hamburg, Deutschland*, [3]*Martin Luther University Halle-Wittenberg, Germany, Halle-Wittenberg, Deutschland*, [4]*Department of General, Visceral, and Thoracic Surgery, University Medical Center Hamburg-Eppendorf, Hamburg, Deutschland*, [5]*Department of Pediatric Surgery, Hauner Children's Hospital LMU Munich, München, Deutschland*

**Background:**

Simulation-based training has become essential in medical education for developing both technical and non-technical competencies. However, barriers such as limited access to expert instructors, resource constraints, and restricted autonomy over educational opportunities often prevent students from acquiring adequate surgical training. Peer-teaching has emerged as a potential solution to these limitations, offering a cost-effective, scalable, and student-centered approach. This study investigates the effectiveness of a student-led laparoscopic simulation training, focusing on the impact of peer-teaching on the skill development, confidence, and career motivations of 65 medical students.

**Materials and methods:**

A structured laparoscopic training program was implemented using Kern's six-step model for curriculum development. Peer instructors guided participants through fundamental laparoscopic skills, including camera navigation, tissue manipulation, and intracorporeal suturing. The training was conducted in a student-led simulation laboratory, utilizing commercially available box trainers. Over the course of nine months two evaluations were performed using pre- and post-training questionnaires, assessing satisfaction, stress levels, preparedness for clinical rotations, and motivation to pursue a career in surgery. On a scale of 1 to 5, students rated key metrics as insignificant or absent (0) to very significant or very present (5). The survey also measured further interest in laparoscopic training and students' perceptions of peer-teaching as a motivating factor in pursuing a surgical career.

**Results:**

The program received overwhelmingly positive feedback from the participants in the first evaluation ($n$=41), including high satisfaction (M = 4.88). Overall, there was a high level of interest in continuing laparoscopic training and a high level of recommendation of surgical training to fellow students (M = 4.88 and M = 4.98). Participants in the second survey ($n$=24) were again very satisfied with the laparoscopic training (M = 4.57) and showed great interest in further and advanced courses (M = 4.43 and M = 4.36). They felt better prepared for future clinical rotations (M = 4.21) and appreciated the peer teaching concept (M = 4.43), with the peer teachers being rated as sufficiently competent in basic laparoscopy (M = 4.43). Participants also felt more comfortable asking questions to the peer teachers compared to surgeons (M = 3.93), but some expressed a desire for more involvement from healthcare professionals. In addition, most participants indicated that they would recommend the training to their colleagues (M = 4.43). Importantly, participants showed a consistent or increased motivation to pursue a career in surgery after the course (M = 4.00).

**Conclusion:**

Our findings demonstrate that peer-teaching, integrated within a student-led surgical simulation laboratory, offers an effective and scalable approach to enhancing both technical skills and enthusiasm for surgical careers among medical students. With a cohort of 65 participants within nine months, this study underscores the potential of peer-driven models in addressing key challenges in surgical education, such as instructor shortages and resource limitations. By fostering confidence and long-term interest in surgery, peer-teaching holds promise for widespread adoption across medical schools, particularly in resource-limited environments. Ultimately, this model could contribute to alleviating workforce shortages by motivating more students to pursue surgical specialties.

**Reporting of Major Complications in Randomized Controlled Trials in Pancreatic Surgery According to Clavien-Dindo Classification – A Systematic Review and Meta-Analysis**
ID: 230

**Kategorie: DGCH - Qualitätsindikatoren in der Chirurgie**

Amila Cizmic[1], Laetitia Hampe[1], Philipp A Wise[2], Pascal Probst[3], Markus K Müller[3], Anna Nießen[1], Faik G Uzunoglu[1], Markus W Büchler[4], Thilo Hackert[1], Felix Nickel[1]

[1]*Department of General, Visceral and Thoracic Surgery, University Medical Center Hamburg-Eppendorf, Hamburg, Deutschland*, [2]*Department of Neuroradiology at the Neurology Center, Heidelberg University Hospital, Heidelberg, Deutschland*, [3]*Department of Surgery, Cantonal Hospital Thurgau, Frauenfeld, Schweiz*, [4]*Botton- Champalimaud Pancreatic Cancer Research Center, Champalimaud Foundation, Lisboa, Portugal*

**Background:**
Standardized reporting of postoperative complications in randomized controlled trials (RCTs) in pancreatic surgery is crucial for objective and reproducible outcome interpretation. Clavien-Dindo Classification (CDC) was introduced to overcome the inconsistencies in postoperative complications reporting. Many RCTs use major complications (MC), according to the CDC, as their primary or secondary endpoint. This study evaluated the current standard of MC reporting in RCTs in pancreatic surgery.

**Materials and methods:**
A systematic review and meta-analysis of RCTs in pancreatic surgery were performed according to the recommendations of the Cochrane Handbook for Systematic Reviews and Interventions and PRISMA guidelines. Two independent researchers identified relevant studies through a systematic search using the Evidence Map of Pancreatic Surgery platform with a systematic search up until the 31st of August 2024. All RCTs that defined MC according to the CDC were included in the study. Further RCTs were screened for reporting all postoperative complications for the meta-analysis, according to CDC.

**Results:**
Of 1066 studies on pancreatic surgery, 366 were identified as RCTs. Sixty-four studies defined MC according to the CDC and included 10984 patients. There was significant heterogeneity in the definition and reporting of MC. Most studies defined MC as CDC ≥ III (n=50, 78.1%). Five (7.8%) studies used only CDC III-IV as MC. Four studies (6.3%) used CDC ≥ IIIb, and one (1.6%) study used CDC > IIIb. The CDC ≥ II was used two times (3.1%) and CDC II-IV twice (3.1%) as the definition of the MC. However, only twelve (18.8%) of the 64 included studies reported all CDC complications. An additional seven RCTs reported all CDC complications, according to the CDC, without defining MC.

**Conclusion:**
The majority of RCTs in pancreatic surgery defined MC as CDC ≥ III. However, there was heterogeneity in that MC in some studies started with CDC II, some started with CDC IIIb or CDC V was not included. This variability limits the comparability and benchmarking of studies and may result in misleading analyses. Therefore, a standardized definition of MC is urgently needed to ensure consistency and reliability in future surgical research. We suggest to define MC in pancreatic surgery as CDC IIIa-V.

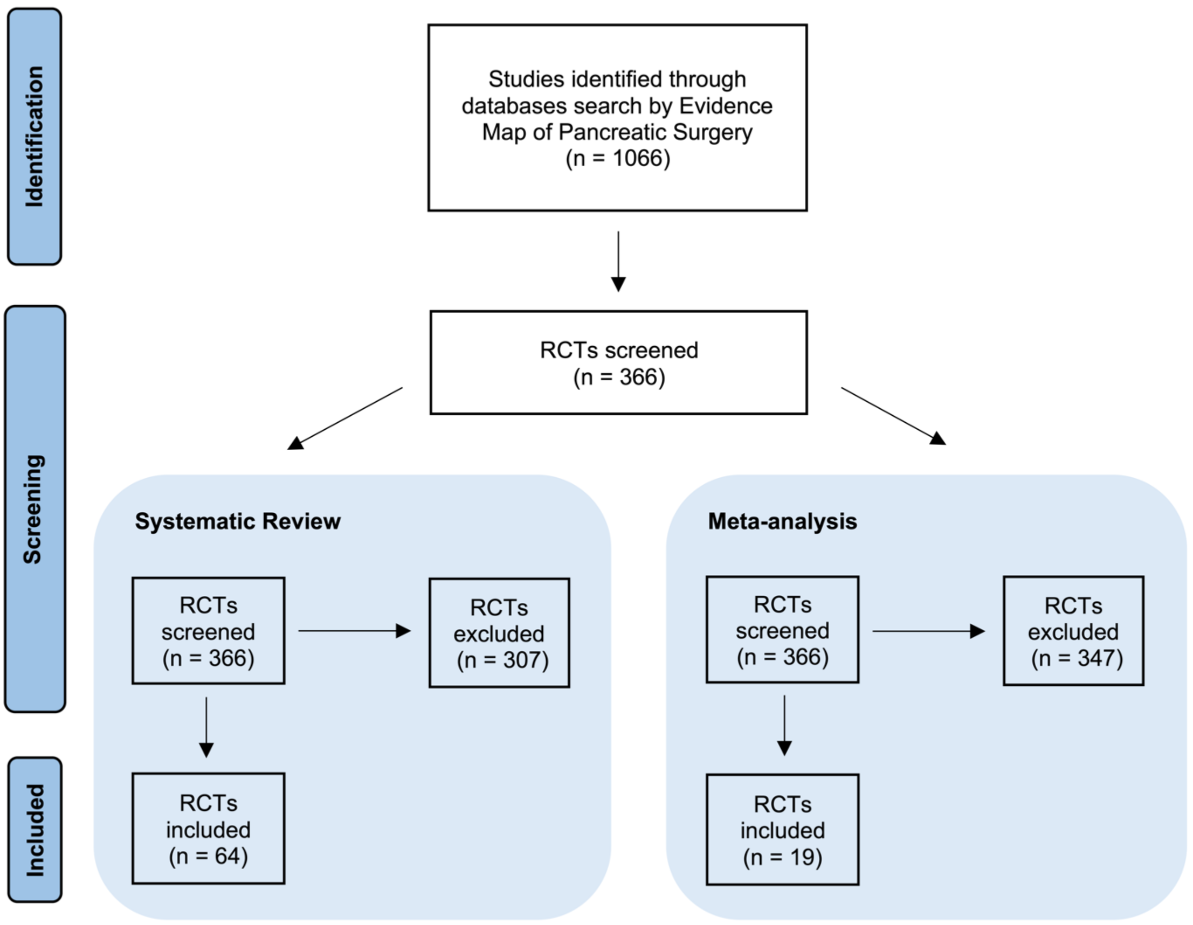

**Figure 1** PRISMA Flow Chart of the Study.

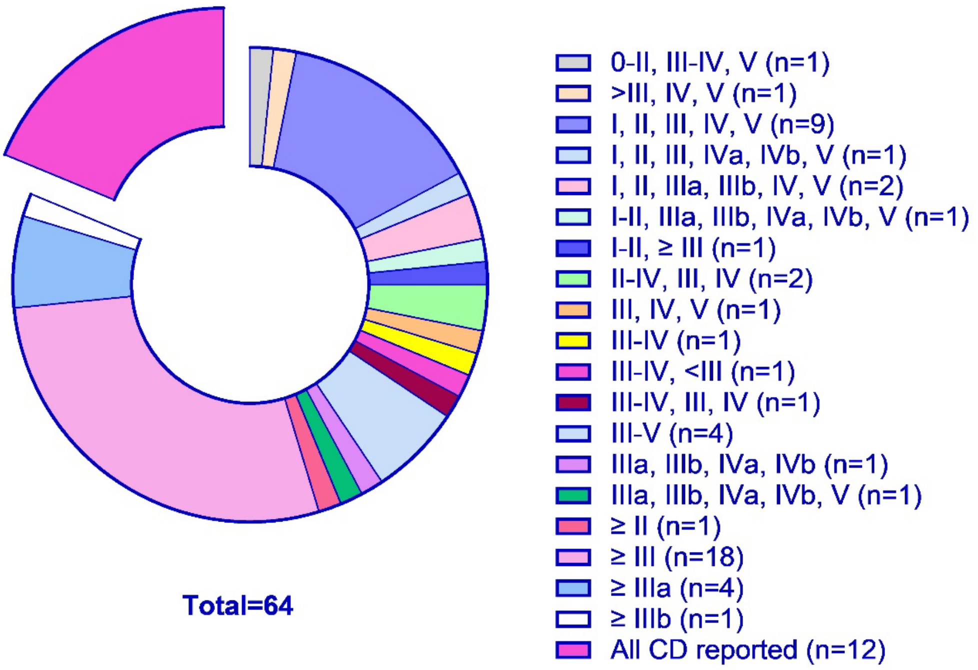

**Figure 2** Observed variations (n = 20) of CDC reporting in the included studies.

Abstracts – DCK 2025 – 142nd Congress of the German Society of Surgery – Munich, March 26–28 • DOI 10.1515/iss-2025-2002
Innov Surg Sci 2025; 10 (Special Suppl 1): s40–s112

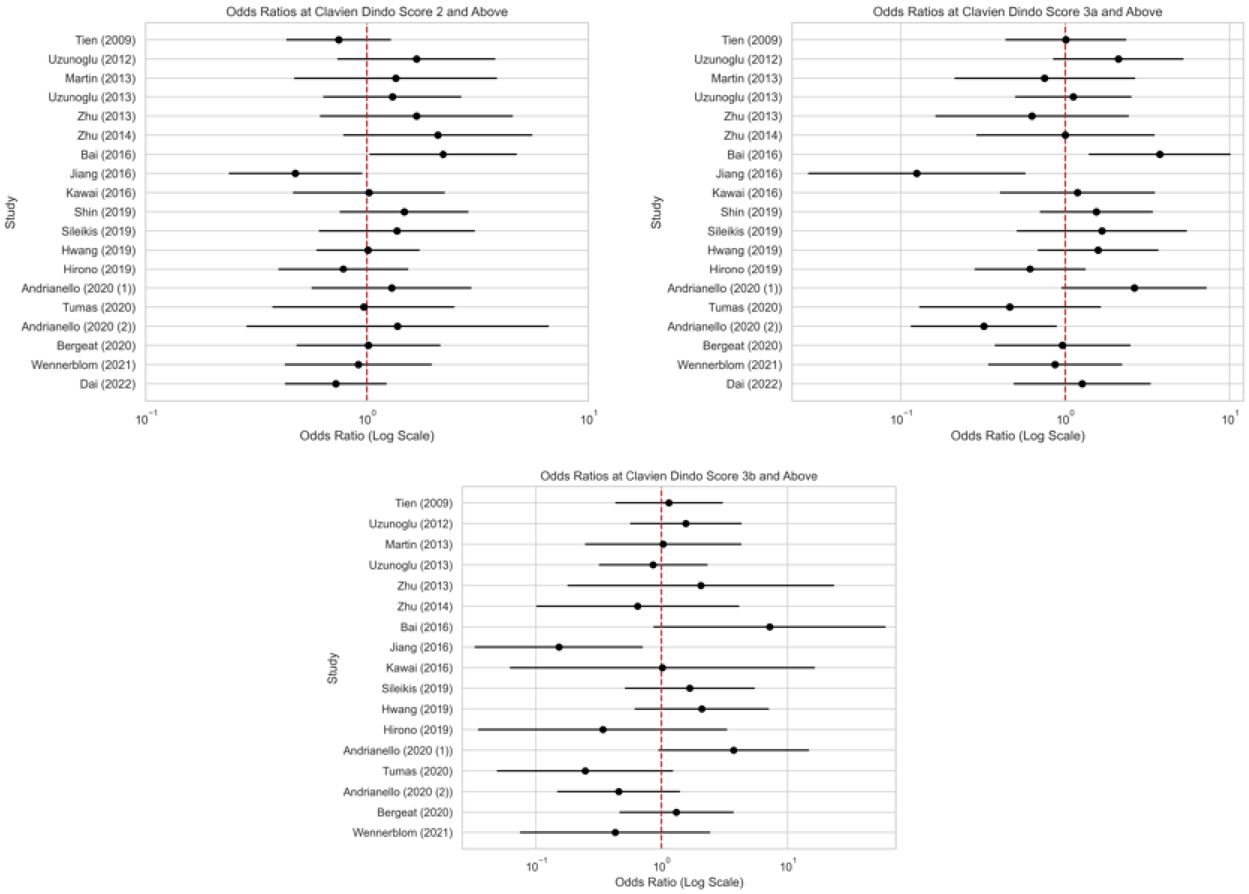

**Figure 3** Forest plots showing odds ratios of postoperative complications for intervention vs. control groups, stratified by Clavien-Dindo score thresholds (≥ II, ≥ IIIa, and ≥ IIIb).

### Bacterial Colonization in Resected Lymph Nodes During Pancreatic Surgery

ID: 420

**Kategorie: DGCH - Qualitätsindikatoren in der Chirurgie**

Saadet Coskun Gürcinar[1], Elke Demir[1], Carsten Jäger[1], Helmut Friess[1], Ihsan Ekin Demir[1]

[1]*Technische Universität München , Klinikum rechts der Isar, Chirurgie, München*

**Background:**
Pancreatic resection remains the primary curative option for localized pancreatic cancer and chronic pancreatitis. However, postoperative complications such as pancreatic fistula, infections, and abscesses continue to pose significant risks. Bacterial colonization and infection of peripancreatic collections are contributing factor sto these complications, although the precise determinants of colonization, i.e., whether they are related to surgical techniques, the tumor microenvironment, or patient-specific factors remain unclear.This study aimed to investigate the prevalence of bacterial colonization in lymph nodes (station 8) resected during pancreatic surgery and its association with postoperative outcomes. Specifically, we evaluated whether bacterial colonization was influenced by surgical techniques, tumor-associated microbiota, or patient-specific factors such as age, BMI, and comorbidities.

**Materials and methods:**
An observational study was conducted on 22 patients undergoing pancreatic surgery between 2019 and 2021. Microbiological assessments of resected lymph nodes were performed using 16S rDNA-PCR to detect bacterial presence. Clinical data, including surgical techniques, histological diagnosis, and comorbidities, were collected. Various statistical analyses were applied to assess associations between bacterial colonization, patient-specific factors, and postoperative outcomes.

**Results:**
Bacterial colonization was detected in 13.6% of cases. No significant correlation was observed between bacterial colonization and surgical techniques ($p = 0.943$) or histological diagnosis ($p = 0.119$). Additionally, bacterial colonization did not significantly impact the severity of postoperative complications, with 86.36% of patients experiencing no clinically relevant postoperative pancreatic fistula.

**Conclusion:**
Bacterial colonization of lymph nodes during pancreatic surgery is relatively uncommon and does not appear to significantly influence postoperative outcomes. These findings suggest that other factors, such as perioperative management and patient-specific risk profiles, may play a more critical role in determining surgical outcomes. Further research is warranted to explore the clinical significance of bacterial colonization and its contribution to postoperative complications.

**Routine, comprehensive measuring of health-related Quality of Life (hrQoL) in pediatric surgery with PedsQL 4.0 Generic Module (PedsQL-GM) and Family Impact Module (PedsQL-FIM) to prepare for hrQoL-based patient management**
ID: 542

**Kategorie: DGCH - Qualitätsindikatoren in der Chirurgie**

Jurek Schultz[1], Anja Zschieschang[1], Henrike Weiß[1], Hannah Stolzenburg[1], Clara Spiller[1], Kevin Herbrig[1], Guido Fitze[1]

[1]*Technical University of Dresden, Pediatric Surgery, Dresden, Deutschland*

**Background:**
PROMs are established for research and should become essential for immediate patient management. However, the age variance that partly necessitates proxy measures, the variety of diagnoses, and the patient-caregiver interplay remain challenging in children. The PedsQL-General Module (GM) is a generic, 23-item, four-dimensional, self- and proxy measure validated in German for children from 0 to 18. The 36-item PedsQL-Family Impact Module (FIM) encompasses eight scales measuring parent and family functioning. We tested the implementation of both instruments for routine, real-time measurements, enabling hrQoL-based patient management.

**Materials and methods:**
Tablet-based PedsQLTM-GM and -FIM were validated and embedded in our hospital software to visualize answers, calculated scores, and references. After educating our personnel, we rolled out PedsQL's routine and comprehensive use. We quantified the time needed for the administration, the time required to answer, return quotas, reasons for unreturned measures, and costs for equipment. A qualitative evaluation addressed problems and situations when PedsQLTM values prompted individual interventions. Psychometric properties were calculated using SPSS and Microsoft Excel.

**Results:**
Starting in 10/2022, we successively enrolled seven clinics. By 04/2024, we evaluated 12,991 questionnaires. Reasons for unreturned questionnaires (16%) included language barriers (50%), patients not interested (12%), not willing to participate again (8%), and not enough staff (6%). In the severely disabled, inappropriate items frequently prompted refusal. The average completion time was 7, respectively, 11 minutes when self-reports were included for older children. Administration time averaged 86 seconds for repeated and 109 seconds for initial handouts. Equipment was minimal (4 tablets/clinic). Overall, the PedsQLTM-GM showed good consistency. Regarding the different dimensions, we saw satisfactory to good consistencies with two exceptions: items in proxy measures for ages 5-7 and 8-12 showed McDonald's Omega of <0.5 in the social dimension. Overall, the PedsQLTM-FIM showed good and, within sub-scales, satisfactory to good internal consistencies.

**Conclusion:**
Our study shows that digital administration of PedsQLTM-GM and -FIM allows for routine, comprehensive, real-time evaluation of hrQoL in most pediatric outpatient diagnoses with good reliability and immediate visibility to staff. This enables hrQoL-based individual interventions at low financial and personal costs. Detailed qualitative evaluations and diagnosis-dependent validations might lead to disease-specific modules, short forms, improved research, and better individual patient care.

### Registry based analysis of cholecystectomy at a German University Hospital
ID: 925

**Kategorie: DGCH - Qualitätsindikatoren in der Chirurgie**

Kira Steinkraus[1 2], Benjamin Mayer[2], Nuh Rahbari[2], Marko Kornmann[2], Benjamin Müssle[2]

[1]*Universitäsklinikum Hamburg-Eppendrof, Hamburg, Deutschland*, [2]*Universitätsklinikum Ulm, Ulm, Deutschland*

**Background:**
Laparoscopic cholecystectomy (LC) is the gold standard for the therapy of symptomatic cholecystolithiasis and acute cholecystitis. The aim of this study was to analyze the surgical approach and outcomes of cholecystectomy (CE) at a university hospital and the comparison with published national data.

**Materials and methods:**
We conducted a retrospective study of all patients undergoing CE at our center between January 2008 and December 2017. Logistic regression analysis was performed to identify risk factors for conversion. Surgical management and complication rates were compared with nationwide annual reports.

**Results:**
A total of 1,537 CEs were included in the analysis. Of these, 881 (57.4%) were elective and 656 (42.7%) were emergency operations. Minimally invasive operations were performed in 1,261 patients (82.2%), the conversion rate was 10.7% (n = 135). In the multivariate regression analysis, preoperative bile duct intervention ($p < 0.001$), previous abdominal surgery ($p = 0.002$), and male gender ($p = 0.011$) were identified as independent risk factors for conversion. Compared to published data, we performed more primary open operations (17.9% vs. 5.0–6.8%), conversion rates (10.7% vs. 4.1–4.7%), postoperative complications (7.8% vs. 2.0–2.1%), and reinterventions (4.3% vs. 2.0–2.6%) were higher. Comparison of patient characteristics revealed that our collective had a significantly higher ASA score (ASA III: 38.6% vs. 22.6–22.8%; ASA IV: 6.3% vs. 1.9%) and were operated more frequently because of acute cholecystitis (65.6% vs. 33.8–37.2%). Mortality (1.3% vs. 0.9–1.0%) and mean length of hospital stay (6.4 days vs. 4.7–5.2 days) were comparable.

**Conclusion:**
A cholecystectomy can be performed at a university hospital with comparable mortality and length of stay in patients with higher morbidity. The data underscores the need for a patient tailored surgical approach in high-risk patients.

**Influence of Certification Program on Treatment Quality and Survival for Rectal Cancer Patients in Germany: Results of 13 Certified Centers in Collaboration with AN Institute**
ID: 957

**Kategorie: DGCH - Qualitätsindikatoren in der Chirurgie**

Mihailo Andric[1] [1], Jessica Stockheim[1], Mirhasan Rahimli[1], Sara Al-Madhi[1], Sara Acciuffi[1], Maximilian Dölling[1], Roland S. Croner[1], Aristotelis Perrakis[1]

[1]*Universitätsklinikum Magdeburg, Universitätsklinik für Allgemein-, Viszeral-, Gefäß- und Transplantationschirurgie, Magdeburg, Deutschland*

**Background:**
The certification of oncological units as colorectal cancer centers (CrCCs) has been proposed to standardize oncological treatment and improve the outcomes for patients with colorectal cancer (CRC). The proportion of patients with CRC in Germany that are treated by a certified center is around 53%. Lately, the effect of certification on the treatment outcomes has been critically discussed. Our aim was to investigate the treatment outcomes in patients with rectal carcinoma at certified CrCCs, in German hospitals of different medical care levels.

**Materials and methods:**
We performed a retrospective analysis of a prospective, multicentric database (AN Institute) of adult patients who underwent surgery for rectal carcinoma between 2002 and 2016. We included 563 patients from 13 hospitals of different medical care levels (basic, priority, and maximal care) over periods of 5 years before and after certification.

**Results:**
The certified CrCCs showed a significant increase in the use of laparoscopic approach for rectal cancer surgery (5% vs. 55%, $p < 0.001$). However, we observed a significantly prolonged mean duration of surgery in certified CrCCs (161 Min. vs. 192 Min., $p < 0.001$). The overall morbidity did not improve (32% vs. 38%, $p = 0.174$), but the appearance of postoperative stool fistulas decreased significantly in certified CrCCs (2% vs. 0%, $p = 0.036$). Concerning the overall in-hospital mortality, we registered a positive trend in certified centers during the five-year period after the certification (5% vs. 3%, $p = 0.190$). The length of preoperative hospitalization (preop. LOS) was shortened significantly (4.71 vs. 4.13 days, $p < 0.001$), while the overall length of in-hospital stays was also shorter in certified CrCCs (20.32 vs. 19.54 days, $p = 0.065$). We registered a clear advantage in detailed, high-quality histopathological examinations regarding the N, L, V, and M.E.R.C.U.R.Y. statuses. In the performed subgroup analysis, a significantly longer overall survival after certification was registered for maximal medical care units ($p = 0.029$) and in patients with UICC stage IV disease ($p = 0.041$). In patients with UICC stage III disease, we registered a slightly non-significant improvement in the disease-free survival (UICC III: $p = 0.050$).

**Conclusion:**
The results of the present study indicate an improvement in terms of the treatment quality and outcomes in certified CrCCs, which is enforced by certification-specific aspects such as a more differentiated surgical approach, a lower rate of certain postoperative complications, and a multidisciplinary approach. Further prospective clinical trials are necessary to investigate the influence of certification in the treatment of CRC patients.

**Does Nephrectomy Increase the Risk of Post-Operative Dialysis in Retroperitoneal Sarcoma Surgery? - A rapid review**
ID: 979

**Kategorie: DGCH - Qualitätsindikatoren in der Chirurgie**

Georgios Polychronidis[1], Sakher Shraim[1], Cosima Engerer[1], Andreas Brandl[1], Mohammed Al-Saeedi[1]

[1]*Universitätsklinikum Heidelberg, Chirurgische Klinik, Heidelberg, Deutschland*

**Background:**
To assess the impact of nephrectomy on the risk of post-operative dialysis in patients undergoing surgery for retroperitoneal sarcomas.

**Materials and methods:**
A rapid review of the literature was performed to identify studies reporting on the incidence of post-operative dialysis in patients undergoing nephrectomy or kidney-preserving surgery for retroperitoneal sarcomas. The data was extracted from the full texts of the studies, and a summary table and forest plot were created.

**Results:**
A total of 10 studies were included in the review. The incidence of post-operative dialysis was low in both groups, with only 13 cases reported across all studies. The pooled analysis did not reveal a statistically significant difference in the odds of requiring dialysis between the two groups.

**Conclusion:**
The available evidence suggests that the risk of post-operative dialysis is low in patients undergoing surgery for retroperitoneal sarcomas, regardless of whether they undergo nephrectomy or kidney-preserving surgery.

### Identifying patients at risk for surgical site infections by reducing complexity of intestinal microbiota with enterosignatures

ID: 204

**Kategorie: DGCH - Risikomanagement in der Chirurgie**

Simone Zwicky[1], Daniel Spari[1], Daniel Rodjakovic[1], Hugo Armando Guillen-Ramirez[1], Bahtiyar Yilmaz[1], Guido Beldi[1]

[1]*Department of Visceral Surgery and Medicine, Inselspital, Bern University Hospital, Bern, Schweiz*

**Background:**
The relationship between preoperative intestinal microbiota composition and the development of SSIs following abdominal surgery is not well understood. Therefore, we characterized the preoperative rectal microbiota using the novel concept of enterosignatures (ESs) in patients undergoing abdominal surgery and tested their association with surgical site infections (SSIs).

**Materials and methods:**
In this prospective cohort study, preoperative rectal microbiota from 133 patients undergoing abdominal surgery were profiled using 16S rRNA sequencing. Enterosignatures were calculated using high-quality genus-level taxonomy, simplifying complex microbial compositions into five generalizable patterns: *Bacteroides, Firmicutes, Prevotella, Bifidobacterium*, or *Escherichia*-dominated profiles.

**Results:**
A total of 519 bacterial species were identified within the 133 patients. The Firmicutes ES was found to be a significant risk factor for SSIs, while the Prevotella ES was associated with a reduced risk of SSIs. Combining these into the Firmicutes-to-Prevotella ES ratio (ES-Firm-Prev ratio) yielded a stronger association with SSIs (median [IQR] log ES-Firm-Prev ratio: no SSI, 0.21 [-0.43, 1.33] vs. SSI, 8.24 [2.17, 8.5]; p = 0.001). Machine learning and logistic regression models confirmed the ES-Firm-Prev ratio as a significant predictor of SSIs, independent of clinical covariates (OR, 1.39; 95% CI, 1.08-1.78; p = 0.01).

**Conclusion:**
The ES-Firm-Prev ratio is a robust, independent predictor of SSIs in patients undergoing abdominal surgery and may serve as a novel biomarker to identify high-risk patients preoperatively.

**The Five-Item Modified Frailty Index (mFI-5) Predicts Adverse Surgical Outcomes in Patients Undergoing Mastectomy**
ID: 211

**Kategorie: DGCH - Risikomanagement in der Chirurgie**

Laetitia Chiarella[1 2 3], Lioba Hülsbömer[3], Fortunay Diatta[3], Felix J. Klimitz[3 4], Alexander J. Kammien[3], Alejandro Kochen[3], Sam Boroumand[3], Omar Allam[3], Martin Kauke-Navarro[3], Michael Alperovich[3], Bohdan Pomahac[3]

[1]*Institut für Muskuloskelettale Medizin , Uniklinikum Münster, Münster, Deutschland*, [2]*Fachklinik Hornheide, Abteilung für Plastische-, Rekonstruktive und Ästhetische Chirurgie, Münster, Deutschland*, [3]*Division of Plastic & Reconstructive Surgery, Yale New Haven Hospital, Yale School of Medicine, New Haven , Vereinigte Staaten*, [4]*BG Trauma Center Ludwigshafen, Plastic- and Hand Surgery, University of Heidelberg, Department of Hand, Plastic and Reconstructive Surgery, Ludwigshafen, Deutschland*

**Background:**
The 5-item modified Frailty Index (mFI-5) is a clinical tool designed to predict adverse outcomes in patients undergoing various surgical procedures. It assesses five key comorbidities: diabetes, hypertension, congestive heart failure (CHF), chronic obstructive pulmonary disease (COPD), and dependent functional status. (1,2) The mFI-5 is commonly used to evaluate the risk of postoperative complications, extended recovery times, and overall survival, especially in patients with complex health conditions, such as those undergoing oncological or gynecological surgeries. In particular, the tool has proven valuable for predicting surgical risks in elderly patients or those with multiple comorbidities, as it quantifies their vulnerability to complications such as infections, wound issues, and organ dysfunction.

**Materials and methods:**
This study used data from the American College of Surgeons National Surgical Quality Improvement Program (ACS-NSQIP). Data were collected on adult patients who underwent mastectomy procedures between January 2017 and December 2022. Patients were identified using Current Procedural Terminology (CPT) codes. Exclusion criteria included cases with incomplete or incorrect data, non-breast-related diagnoses, concurrent invasive surgeries, and non-elective procedures. Additionally, male and non-binary patients were excluded. This yielded a final cohort of adult female mastectomy patients. Preoperative, perioperative, and 30-day postoperative data were extracted. Preoperative data included patient demographics, comorbidities (e.g., diabetes, COPD, CHF), and functional status, as well as ASA physical status scores and BMI. Perioperative data included the type of anesthesia, surgical setting (inpatient or outpatient), and year of surgery. Postoperative outcomes included surgical complications such as septic shock, myocardial infarction, deep vein thrombosis, and infections. Further outcomes were operative time and length of hospital stay. Data from ACS-NSQIP were transferred to Microsoft Excel and analyzed using STATA16. Kolmogorov-Smirnov tests assessed data distribution. ANOVA or non-parametric tests (Wilcoxon Rank-Sum or Kruskal-Wallis) were used to compare continuous variables, while categorical variables were analyzed with Pearson's Chi-squared test. Logistic and linear regression models, both unadjusted and adjusted for age and BMI, were employed to assess the association between mFI-5 and outcomes. The modified Frailty Index (mFI-5) was dichotomized at a cutoff of 2, and statistical significance was set at $p < 0.05$. The Benjamini-Hochberg procedure was used to adjust for multiple comparisons.

**Results:**
Between 2017 and 2022, 860 patients underwent mastectomy within the Yale Healthcare Network, of whom 163 (19%) had an mFI score ≥2. The average age of the cohort was 58.79 ± 12.60 years, with a mean BMI of 29.36 ± 6.94. Most patients were White (80.35%), and nearly half (48.84%) had an ASA status of 3. Patients with mFI ≥2 were significantly older (mean age 66.32 ± 10.83 years) than those with mFI <2 (mean age 57.03 ± 12.34 years, p<0.001), and had a higher BMI (33.69 ± 7.73 vs. 28.34 ± 6.34, p<0.001). There were also significant racial differences, with a higher proportion of Black patients in the high-risk group (31.3% vs. 13.5%, p<0.001).

Surgical complications were observed in 11.98% (n=123) of patients. The high-risk frailty group (mFI ≥2) experienced significantly more complications (22.7%, n=37) compared to the low-risk group (9.5%, n=66, p<0.001). In unadjusted multivariate logistic regression, patients with mFI ≥2 were nearly three times more likely to develop complications (OR 2.81; [1.80-4.38], p<0.001). After adjusting for age and BMI, this association remained robust (aOR 2.66; [1.60-4.43], p<0.001).

Although linear regression indicated a slight reduction in hospital stay and surgery duration for the high-risk group (p=0.65) or surgery duration (p=0.33).

As a sensitivity analysis, we performed an adjusted Firth's penalized logistic regression to account for perfect prediction issues that arose from certain components of the composite outcome, 'overall surgical complications,' perfectly predicting the result.
In the adjusted model, several covariates were significantly associated with an increased risk of postoperative complications. Patients with an mFI score of ≥2 had notably higher odds of developing acute kidney failure (aOR 8.43, [2.56–27.83], *p*<0.001), pneumonia (aOR 3.62, [1.33–9.85], *p*=0.012), and deep vein thrombosis (aOR 8.06, [1.95–33.33], *p*=0.004).

**Conclusion:**
In summary, the mFI-5 effectively predicted the risk of surgical complications in mastectomy patients, particularly those with high frailty scores.

(1) Subramaniam S, Aalberg JJ, Soriano RP, Divino CM. New 5-Factor Modified Frailty Index Using American College of Surgeons NSQIP Data. *J Am Coll Surg*. 2018;226(2):173-181.e8. doi:10.1016/j.jamcollsurg.2017.11.005
(2) Dammeyer K, Alfonso AR, Diep GK, et al. Predicting postoperative complications following mastectomy in the elderly: Evidence for the 5-factor frailty index. *Breast J*. 2021;27(6):509-513. doi:10.1111/tbj.14208

**Ventilation systems in the OR: possibilities and limitations in the prevention of surgical site infections**
ID: 238

**Kategorie: DGCH - Risikomanagement in der Chirurgie**

Sebastian Buhl[1], Clemens Bulitta[1]

[1]*Ostbayerische Technische Hochschule Amberg-Weiden, Weiden, Deutschland*

**Background:**
Surgical site infections (SSI) are a known risk factor in many surgical procedures. Although several studies have already shown the impact of air contamination on the risk of infection, the effect of ventilation systems in the OR is still the subject to controversial debate. Despite the sometimes contradictory data, there are already studies that show a direct correlation between the use of a modern ventilation system and the rate of surgical site infections. In principle, these ventilation systems have several purposes and use different modes of action to deliver these functions.

**Materials and methods:**
A recent development in the field of ventilation technology in the operating theater is the so-called temperature-controlled airflow (TcAF) ventilation. The TcAF is a relatively new and innovative concept in ventilation and air conditioning systems. This system controls the temperature of the operating room and thus regulates the temperature of the HEPA-filtered supply air. Similar to a low-turbulence displacement flow, a protected area with ultraclean air is created via outlets above the operating field. However, the contaminated air is displaced by physical effects. The supply air, which is 1.5 Kelvin cooler, sinks downwards at a speed of approx. 0.25 m/s due to the temperature difference, displacing the contaminated room air in the area above the operating table. With a volume flow rate of 350 m³/h per air outlet and 8 outlets, a total volume flow rate of 2,800 m³/h is achieved with the TcAF technology. Together with the company Avidicare, the Ostbayerische Technische Hochschule (OTH) has carried out investigations into the effectiveness and efficiency of such a ventilation system both in the university's own research operating theater and under real conditions in hospitals. These tests were based both on a standardized particle measurement (protection degree) in accordance with DIN 1946-4 and on a check of microbiological air contamination in accordance with SIS-TS 39.

**Results:**
Our results clearly demonstrate the effectiveness and efficiency of the TcAF system. The minimization of the particle and airborne microbial burden in the operating room was proven by both active airsampling measurement and protection level determination. The results are below the limit values for the protection degree according to DIN 1946-4 and the microbiological contamination according to SIS-TS 39. All measurment points delivered favourable results with the ventilation system in the entire protected area. The average values here are between 0 and 2.5 CFU/m³. Also in the tests in the hospitals the germ measurements showed values of median (M) 0 cfu/m³ over all measuring points in the room, mean value (MW) 1.8 cfu/m³, standard deviation (SD) 4.5 cfu/m³.

**Conclusion:**
The use of ventilation systems in the operating theater leads to a reduction in airborne pathogens. Although the germ-reducing effect has already been demonstrated, particularly in systems with a protective zone, the impact of ventilation systems on the rate of SSI remains unclear. One possible explanation could lie in the underlying test procedures used to test the ventilation systems. Here, the effectiveness is often tested under artificial conditions (empty room, no staff, no medical devices). Since the operating room is a complex thermodynamic system that is impacted by many factors, the system should be tested under realistic operational conditions. Another factor is the design of the ventilation system. The impact of ventilation systems on the rate of surgical site infection needs to be further investigated in order to be accurately assessed and to develop and implement optimal ventilation technology solutions.

**Weekday Effect of Index Surgery in Oncological Upper GI resections: A retrospective analysis with prospective data on disease-free survival**
ID: 444

**Kategorie: DGCH - Risikomanagement in der Chirurgie**

Annika Niezold[1], Patrick Sven Plum[1], Daniel Seehofer[1], Konstantin Lukas Uttinger[1 2]

[1]*Universitätsklinikum Leipzig, Leipzig, Deutschland*, [2]*Universitätsklinikum Frankfurt, Frankfurt, Deutschland*

**Background:**
It is unclear if the weekday of esophageal and gastric major resections impacts patient outcome. The aim was to analyze weekday dependency after esophageal and gastric resections with regards to disease-free survival and overall survival.

**Materials and methods:**
This is a retrospective monocentric study with a prospective analysis of cancer recurrence and life status based on patient records undergoing major upper GI surgery with a subsequent malignant diagnosis in the histologic specimen at the University Hospital Leipzig between 2012 and 2021. Procedural (OPS) and diagnosis (ICD) Coding was used to identify patient records undergoing major surgery of the upper GI. Analysis of readmissions with proof of cancer recurrence was performed. In addition, as of March 31$^{st}$ 2024, a life status was obtained using official registry data (“Melderegister”, “Standesamt”). Disease-free survival was defined as primary endpoint. A multivariate Cox-regression model was built using a backfitting elimination method.

**Results:**
In total, 332 patients with major surgery of the upper GI were analyzed, of whom 158 (47.6%) were esophageal and 174 (52.4%) gastric resections. Malignancy was histologically diagnosed in 127 (80.4%) of patients in esophageal and 142 (81.6%) in gastric resections, who were analyzed further. Of these, 19 (15.0%) and 43 (30.3%) were females, respectively. The median age was 60 years in esophageal (interquartile range, IQR, 53 to 69) and 65 years in gastric oncologic resections (IQR 55-74). At the time of resection, the distribution of UICC stages was as follows for esophageal cancer: 0: 14 (13.0%), I: 30 (27.8%), II: 17 (15.7%), III: 34 (31.5%) and IV: 13 (12.0%) and as follows for gastric cancer: 0: 3 (2.3%), I: 44 (33.6%), II: 34 (26.0%), III: 32 (24.4%) and IV: 18 (13.7%). The most common index procedure in esophageal resections was total esophagectomy with restoration of continuity, accounting for 66.1% (84). The most common gastric resection was total gastrectomy without esophageal resection (87, 61.3%). Most esophageal resections were performed Thursday (46, 36.2%), the fewest Monday (9, 7.1%). In gastric surgery, most resections were performed Wednesday (42, 29.6%), and the fewest Monday (15, 10.6%). There were no oncological resections on the weekend. Disease-free survival as primary endpoint was 44.9% after esophageal resection at the end of follow-up (median follow-up: 68.6 months, IQR 13.4-95.2, median time to recurrence or death: 34.8 months, IQR 8.1-85.1). Disease-free survival was 53.5% after gastric resection at the end of follow-up (median follow-up: 50.2 months, IQR 21.1-85.0, median time to recurrence or death: 30.9 months, IQR 9.2-75.8). By weekday of index surgery, disease-free survival was lowest on Monday for esophageal resections (33.3%, median time to recurrence or death 55.3 months, IQR 5.7-97.2), while it was highest on Friday (50.00%, median time to recurrence or death 47.4 months, IQR 4.5-81.5). In gastric resections, disease-free survival was lowest on Monday (40.0%, median time to recurrence or death 75.9 months, IQR 31.3-92.8) and highest on Wednesday (64.3%, median time to recurrence or death 34.1 months, IQR 9.1-81.1). After factor selection using a backfitting elimination method, a Cox regression model adjusted to weekday of surgery, age and UICC stage was used to identify a weekday effect, while comorbidity and extent of main surgery were not included due to insignificance, and sex was only included in gastric resections. In this model, in reference to Friday procedures, procedure performance was not associated with increased risk of cancer recurrence or death on any weekday in esophageal resections (Hazard Ratio, HR, for Monday resections 1.83, 95% Confidence Interval, CI, 0.52-6.43, p=.344). In gastric resections, Tuesday procedures were associated with increased risk of cancer recurrence or death (HR 2.60, 95% CI 1.04-6.48, p=.041) in reference to Wednesday procedures.

**Conclusion:**
In esophageal resections, no weekday effect was observed in this analysis. In gastric resections, Tuesday procedures were associated with increased risk of death or disease recurrence in reference to Wednesday procedures.

**Sealing the Gap: Tackling Iatrogenic Esophageal Perforations via EndoVAC**
ID: 466

**Kategorie: DGCH - Risikomanagement in der Chirurgie**

Jennifer Merten[1], Tariel Stephan Tschaidse[1], Jens Peter Hölzen[1], Mazen Juratli[1], Andreas Pascher[1], Ann-Kathrin Eichelmann[1]

[1]*Universitätsklinikum Münster, Klinik für Allgemein-, Viszeral- und Transplantationschirurgie, Münster, Deutschland*

**Background:**
Due to the lack of serosa, the esophagus is more susceptible to iatrogenic perforations, e.g. during TEE/interventional endoscopies, than the rest of the gastrointestinal tract. These occur very rarely, but are extremely difficult to treat depending on the level of perforation and have a high mortality rate. The European Society of Gastrointestinal Endoscopy recommends endoscopic closure if possible. The advantage is that patients are usually fasting with a consequent lack of contamination of the perforation cavity and rapid diagnosis during the elective examination. Depending on the availability of treatment and expertise, treatment is therefore usually carried out by means of clipping, stenting and, increasingly, endoscopic suturing. Endoluminal vacuum therapy (EndoVAC) is not the first choice in most centres and has been used more for contaminated or chronic perforations so far. This study analyses the outcome of EndoVAC therapy after iatrogenic esophageal perforation.

**Materials and methods:**
All patients were included retrospectively who were referred to our clinic in the period 2013 - 2023 due to iatrogenic esophageal perforation and treated with EndoVAC. The mean follow-up was 10 months.

**Results:**
Within 10 years, 71% of 55 patients (n=39, 67% female, 68 years, BMI 25.7 kg/m²) were treated with EndoVAC due to the extent of perforation. In more than half of these patients (56%), a TEE/interventional endoscopy was the cause. The perforation, which was on average 6 cm long, was predominantly localised in the proximal (= up to 20 cm from the front teeth (46%)) or middle (= up to 30 cm from the front teeth (28%)) esophagus. All patients were treated with antibiotics and almost half of them received chest drainage (56%). In n=5 a combination therapy consisting of esophageal suture and EndoVAC was performed. The patients received an average of 4 VAC cycles (suction -100 to -150mmHg) over 17 days. Intracavitary therapy was performed in n=16 (41%).
In n=8 (20%) a discontinuity resection had to be performed. N=1 underwent palliative stenosis due to tumour perforation and n=7 (18%) died of mediastinitis/sepsis, 2 of them within the first 3 days in hospital. In 59% the iatrogenic esophageal perforation could be healed by EndoVAC therapy.
The hospital stay up to discharge on a soft food was 31 (1-104) days, of which 13 (1-104) days in 87% were spent in an observation/intensive care unit. Pulmonary complications occurred frequently, of which n=23 were pleural effusions or empyema. No endoscopic complications occurred.

**Conclusion:**
Iatrogenic esophageal perforation is a rare but serious complication that is not easy to treat either endoscopically or surgically due to its mostly proximal location.
In our group, we were able to demonstrate healing with EndoVAC therapy in over half of the patients with high esophageal perforation. If there is no response to EndoVAC therapy, a discontinuity resection should be evaluated at an early stage.

**"The Critical 18" of postoperative white blood cells and C-reactive protein predict postoperative pancreatic fistula after distal pancreatectomy**
ID: 616

**Kategorie: DGCH - Risikomanagement in der Chirurgie**

Gwendolin Seidel[1], Ughur Aghamaliyev[1], Yannick Meyer[1], Athanasios Zamparas[1], Bernhard Renz[1], Jens Werner[1]

[1]*Klinikum der Universität München, Klinik für Allgemein-, Viszeral- und Transplantationschirurgie, München, Deutschland*

**Background:**
Distal pancreatectomy (DP) is the primary curative approach for pancreatic ductal adenocarcinoma (PDAC) in the pancreatic tail, and its use has increased due to advances in detecting pancreatic neuroendocrine tumors (pNET) and intraductal papillary mucinous neoplasms (IPMNs). Although DP is less complex than pancreatoduodenectomy (PD), it carries a higher risk of postoperative pancreatic fistula (POPF), a serious complication that can lead to hemorrhage, sepsis, organ failure, and even death. Various risk factors for POPF, including BMI, pancreatic duct diameter, texture, and neck thickness, have been identified. While minimally invasive DP improves quality of life, it does not significantly reduce POPF incidence compared to open surgery. Recent studies, including the PANDORINA trial, have suggested that avoiding intraoperative drain insertion may lower the risk of POPF. As the main diagnostic tool was measurement of drain amylase in order to diagnose POPF, now serum markers are attractive tools to predict POPF. Inflammation parameters, such as CRP and IL-6, have been shown to predict POPF in pancreatoduodenectomy and other abdominal surgeries. This study aims to investigate the predictive value of leukocytes, CRP, and IL-6 for POPF following distal pancreatectomy.

**Materials and methods:**
This retrospective cohort study included patients who underwent DP at a single center between January 2014 and December 2023, with ethics approval. Adult patients who had elective DP were included, while those with prior pancreatic surgery, combined DP and enucleation, or missing data were excluded. Data collected included preoperative demographics, comorbidities, and intraoperative details like surgery type, operative time, blood loss, and resection method. Postoperative parameters included serum markers, such as white blood cells (WBC), C-reactive protein (CRP), and interleukin-6 (IL-6) on POD0 till POD5.

**Results:**
The study comprised 366 patients, who underwent DP between 01/2014 and 12/2023 and met inclusion criteria and were subject of this study. The median age was 65 years (range 18–90), with 186 men (50.8%) and 180 women (49.2%). Postoperatively, median WBC and CRP levels were consistently higher in the group that developed POPF B/C compared to the non-POPF group across the five postoperative days. IL-6 did show no significant predictive value. On POD 2, WBC showed an area under the ROC curve (AUC) of 0.63 ($p < 0.001$), with an optimal cutoff value of 17.9 x 10^9/L, yielding a sensitivity of 52% and a specificity of 72%. Similarly, on POD 3, CRP levels had an AUC of 0.63 ($p < 0.001$), with an optimal cutoff of 17.25 mg/L, resulting in a sensitivity of 64% and a specificity of 62%. Subsequently, 210 patients with available both WBC data on POD 2 and CRP data on POD 3 were selected for further analysis. In the univariate analysis, BMI, WBC >18 x 10^9/L, and CRP >18 mg/dl were identified as independent factors associated with POPF B/C. Multivariate analysis confirmed BMI as an independent risk factor for POPF B/C, with WBC >18 x 10^9/L and CRP >18 mg/dl serving as independent predictive factors for POPF B/C. Among the 210 patients, 102 (48%) had both WBC and CRP levels below the thresholds of 18 x 10^9/L and 18 mg/dl, respectively, and in this subgroup, the rate of POPF B/C was 18%. In cases where CRP levels exceeded 18 mg/dl but WBC remained below 18 x 10^9/L, the POPF rate increased to 37%. When “The Critical 18”, defined as both CRP >18 mg/dl and WBC >18 x 10^9/L, was present, the POPF B/C rate rose to 60%.

**Conclusion:**
In light of these findings, we propose the "The Critical 18", defined as as a simple yet powerful tool for early risk stratification of POPF B/C following distal pancreatectomy. By using WBC >18 x 10^9/L on POD 2 and CRP >18 mg/L on POD 3, clinicians can easily identify patients at escalating risk, offering a clear framework for guiding postoperative management and interventions.

### Enhancing Postsurgical Outcome Predictions Using Explainable AI and Strategic Data Management

ID: 193

**Kategorie: DGCH - Robotische Forschung und KI**

Hugo Armando Guillen-Ramirez[1], Daniel Sanchez-Taltavull[1], Sarah Peisl[1], Stéphanie Perrodin[1], Karen Triep[2], Olga Endrich[2 3], Guido Beldi[1]

[1]*Department of Visceral Surgery and Medicine, Inselspital, Bern University Hospital, University of Bern, Bern, Schweiz*, [2]*Medical Directorate, Medizincontrolling, Inselspital, University Hospital Bern, Insel Gruppe, Bern, Schweiz*, [3]*Department of Clinical Chemistry, Inselspital, Bern University Hospital, University of Bern, Bern, Schweiz*

**Background:**
Infections following surgery drive patient morbidity and mortality, making early detection essential. In the postoperative course, C-reactive protein measurements at postsurgical day 4 have the best predictive power for specific patient populations. Kinetic features, i.e., changes over time or combinations of laboratory parameters, have not been assessed systematically using artificial intelligence in large datasets.

**Materials and methods:**
We have designed and evaluated a workflow to predict postoperative infection independently of the surgical procedure performed, using preoperative, intraoperative, and postoperative characteristics extracted from electronic health records. Electronic health records of 91,794 surgical patients from one large university center were prepared for machine learning approaches. Missing laboratory values were strategically imputed to build a prediction model incorporating both their static and kinetic properties.

**Results:**
The integration of kinetic features of routine laboratory markers in the model revealed that alkaline phosphatase, creatinine, and platelets, in addition to C-reactive protein, improved the predictive ability of the model, achieving a high sensitivity above 0.7. This performance outperformed static C-reactive protein measurements at postoperative days 3 and 4. Interestingly, the study showed that neutrophil levels were not relevant for the prediction of postoperative infections.

**Conclusion:**
We have developed an explainable artificial intelligence model that predicts postoperative infections earlier than existing standard predictive models. The model's transparency enhances its potential for adoption in clinical practice, as clinicians are more likely to integrate novel tools when they can understand and trust the underlying decision-making process.

**Benchmarking vision capabilities of large language models in surgical examination questions**
ID: 249

**Kategorie: DGCH - Robotische Forschung und KI**

Jean-Paul Bereuter[1], Mark Enrik Geissler[1], Anna Klimova[2], Robert-Patrick Steiner[3], Kevin Pfeiffer[4], Fiona Kolbinger[1 5], Isabella Wiest[4 6], Hannah Sophie Muti[1 4 7], Jakob Nikolas Kather[4 7 8]

[1]*Department of Visceral, Thoracic and Vascular Surgery, Faculty of Medicine and University Hospital Carl Gustav Carus, TUD Dresden University of Technology, Dresden, Deutschland*, [2]*Institute for Medical Informatics and Biometry, Faculty of Medicine and University Hospital Carl Gustav Carus, TUD Dresden University of Technology, Dresden, Deutschland*, [3]*Institute of Pharmacology and Toxicology, Faculty of Medicine and University Hospital Carl Gustav Carus, TUD Dresden University of Technology, Dresden, Deutschland*, [4]*Else Kroener Fresenius Center for Digital Health, Faculty of Medicine and University Hospital Carl Gustav Carus, TUD Dresden University of Technology, Dresden, Deutschland*, [5]*Weldon School of Biomedical Engineering, Purdue University, West Lafayette, United States Minor Outlying Islands*, [6]*Department of Medicine II, Medical Faculty Mannheim, Heidelberg University, Mannheim, Deutschland*, [7]*Medical Oncology, National Center for Tumor Diseases (NCT), University Hospital Heidelberg, Heidelberg, Deutschland*, [8]*Department of Medicine I, Faculty of Medicine and University Hospital Carl Gustav Carus, TUD Dresden University of Technology, Dresden, Deutschland*

**Background:**
Recent studies investigated the potential of large language models (LLMs) for clinical decision making and answering exam questions based on text input. Recent developments of LLMs have extended these models with vision capabilities (vision-language models, VLMs). However, there is limited investigation on the applicability of VLMs and their capabilities of answering exam questions with image content. Therefore, the aim of this study was to examine the performance of publicly accessible LLMs in three different surgical question sets consisting of text and image questions.

**Materials and methods:**
Original text and image exam questions from two different surgical question subsets from the German Medical Licensing Examination (GMLE) and United States Medical Licensing Examination (USMLE) were collected and answered by publicly available LLMs (GPT-4, Claude-3 Sonnet, Gemini-1.5). LLM outputs were benchmarked for their accuracy in answering text and image questions. Additionally, the LLMs' performance was compared to students' performance based on their average historical performance (AHP) in these exams. Moreover, variations of LLM performance were analyzed in relation to question difficulty and respective image type.

**Results:**
Overall, all LLMs achieved scores equivalent to passing grades (> 60%) on both surgical text question datasets. On image-based questions, only GPT-4 exceeded the score required to pass, significantly outperforming Claude-3 and Gemini-1.5 (GPT-4: 78% vs. Claude-3: 58% vs. Gemini-1.5: 57.3%; $p < 0.001$). Additionally, GPT-4 outperformed students on both text (GPT-4: 83.7% vs. AHP students: 67.8%; $p < 0.001$) and image questions (GPT-4: 78% vs. AHP students: 67.4%; $p < 0.001$).

**Conclusion:**
GPT-4 demonstrated substantial capabilities in answering surgical text and image exam questions. Therefore, it holds considerable potential for the use in surgical decision making and education of students and trainee surgeons.

**AI-based algorithm for intraoperative organ identification based on hyper spectral imaging**
ID: 286

**Kategorie: DGCH - Robotische Forschung und KI**

Jan Bardenhagen[1], Felix Nickel[1], Thilo Hackert[1], Valerie Isabel Nottberg[1]

[1]*Klinik für Allgemein-, Viszeral- und Thoraxchirurgie - Universitätsklinikum Hamburg-Eppendorf - UKE, Hamburg, Deutschland*

**Background:**
Hyperspectral imaging (HSI) is emerging as a transformative technology in surgery, offering precise tissue identification and real-time assessment of tissue quality. Unlike conventional imaging methods, HSI captures a wide spectrum of light and data (550-995nm), providing detailed insights into physiological parameters such as oxygen saturation and perfusion in a non-invasive manner. Recent applications of HSI in neurosurgery, visceral surgery, and reconstructive procedures have highlighted its potential in evaluating tissue viability and informing surgical decisions. However, current research on visceral surgery is largely limited to animal models. Simultaneously, artificial intelligence (AI) and big data analysis allow sufficient real time-analysis of images and large data collections. this study we aim to explore if an AI-based model is capable of sufficient analysis of images created by HSI.

**Materials and methods:**
Hyperspectral imaging (HSI) was conducted intraoperatively during major abdominal surgeries, including pancreatic, hepatobiliary, upper gastrointestinal, and colorectal procedures. The imaging was performed at different time points depending on the specific type of surgery. The acquired hyperspectral images were then annotated with the corresponding anatomical structures to create a comprehensive dataset for training an AI-based algorithm aimed at tissue identification.
Instance segmentation was implemented using convolutional neural networks (CNN) such as YOLO (You Only Look Once), and different models were developed and compared. Statistical analysis included both descriptive and inferential methods, with model performance evaluated using metrics such as accuracy, precision, sensitivity (recall), mean Average Precision (mAP), and the F1-score. Model validation was performed through k-fold cross-validation to ensure robust estimation of model quality across different subsets of the data.

**Results:**
A total of 243 hyperspectral images were acquired and annotated, covering various classes, including the liver, pancreas, small intestine, large intestine, stomach, esophagus, duodenum, mesentery, adipose tissue, surgical instruments, and sponges. In total, 511 structures were labeled, with "liver" being the most frequently represented (111 instances) and "stomach" the least (21 instances). Each tissue class exhibited distinct hyperspectral properties in terms of parameters like tissue water content, oxygen saturation, and hemoglobin concentration, facilitating their identification.
The AI-based model was trained by using the labeled images. To prevent overfitting and improve model generalizability, regularization techniques like data augmentation (including rotations, scaling, and shifting) were employed. After training, the model was able to recognize different tissues based on their unique HSI parameters. Notably, after training the algorithm could identify different structures and their borders relative to other organs without the need for prior annotation. The specificity of tissue identification ranged from 0.43 for the stomach to 0.92 for surgical instruments. Higher specificity was associated with structures that had a greater number of instances in the dataset, while tissues with lower representation showed reduced specificity, highlighting the influence of dataset composition on the algorithm's accuracy.

**Conclusion:**
The study demonstrates that integrating hyperspectral imaging (HSI) with AI-based algorithms can effectively differentiate various tissue types during major abdominal surgeries. However, specificity varies depending on tissue representation in the training dataset. More commonly encountered structures, like the liver, showed higher specificity, while less frequent tissues, such as the stomach, posed identification challenges, underscoring the need for diverse and comprehensive datasets to enhance AI accuracy.
HSI provides functional imaging data beyond superficial characteristics, capturing physiological parameters like oxygen saturation and tissue water content. Combined with AI, this technology offers potential for more precise intraoperative detection of malignant tissue and areas of hypoperfusion, informing surgical decisions and reducing postoperative complications.
The results, while promising, indicate the necessity of further research and larger datasets to improve the algorithm's performance and clinical applicability. Expanding the HSI image database across various tissue types will refine AI-driven tissue identification. In the future, the integration of HSI and AI may become a powerful tool for real-time, precision-guided surgical navigation, potentially transforming the standard of care in visceral and other surgical fields.

### Towards automated surgical phase recognition in robot-assisted right-sided hemicolectomy using machine learning

ID: 296

**Kategorie: DGCH - Robotische Forschung und KI**

Ferdinand Schweigert[1], André Schulze[1 2 3], Antonia Kraneis[1], Sebastian Bodenstedt[3 4 5 6], Alexander C. Jenke[4 5 6], Marius Distler[1 2 3], Stefanie Speidel[3 4 5 6], Jürgen Weitz[1 2 3], Martin Wagner[1 2 3]

[1]*Department of Visceral-, Thoracic- and Vascular Surgery, University Hospital Carl Gustav Carus, Technical University Dresden, Dresden, Deutschland*, [2]*National Center for Tumor Diseases (NCT/UCC), Dresden, Deutschland*, [3]*Center for the Tactile Internet with Human in the Loop (CeTI), Technical University Dresden, Dresden, Deutschland*, [4]*Department of Translational Surgical Oncology, National Center for Tumor Diseases, Dresden, Deutschland*, [5]*German Cancer Research Center (DKFZ), Heidelberg, Deutschland*, [6]*Helmholtz-Zentrum Dresden-Rossendorf (HZDR), Dresden, Deutschland*

**Background:**
The prevalence of colon cancer is projected to increase by more than 50% in 2040 compared to 2020 and surgical resection is a mandatory part of curative treatment.
Improvement in surgical skill and quality have shown to have an impact on patient outcomes, their assessment may help in training or quality improvement programs. Here, Artificial Intelligence may help to scale the previously manual assessment. For example, workflow analysis such as phase recognition could highlight specific areas for improvement. However, currently there are few studies focusing on automated surgical phase recognition in colorectal surgery.
The study aims to apply a machine learning (ML) model for automatic surgical phase recognition in right-sided hemicolectomy to correctly identify different phases as an important step towards objective surgical quality assessment.

**Materials and methods:**
We collected 24 videos of robot assisted minimally invasive right-sided hemicolectomy from the University Hospital Dresden performed between 2018 and 2024.
Our workflow model consists of seven phases derived from the RIGHT Study from the University Hospital Amsterdam, which used a Delphi process to define the phase model. The seven phases are (1) Setup and exposure of operating field, (2) Submesenteric/ subileal dissection, (3) Superior mesenteric vein (SMV) dissection and ligation of ileocolic vessels, (4) Proximal dissection of the SMV and dissection of the Gastrocolic Trunk of Henle, (5) a) Ligation of the right branches of the middle colic vessels, b) Ligation of middle colic vessels in cases of transverse colon tumour, (6) Hepatic flexure mobilization and colon and ileum transection and (7) Anastomosis. We decided to add two phases: phase 0 as an exceptional phase for parts which are not part of the initial surgery and a phase 8 as the final overview of the procedure. The videos have been annotated by two specifically trained medical students according to a predefined annotation protocol based on the RIGHT study. After annotation both raters compared their annotations and formed a consensus agreement.
In order to automate surgical workflow analysis based on our phase model, we applied the ML algorithm Temporal Convolutional Network for the Operating room (TeCNO), which is a multi-stage learning approach.

**Results:**
Mean operation time was 207 minutes, with a median of 200 minutes and a standard deviation of 69 minutes. When examining the time taken to perform each phase, the longest average duration was observed in phase 2, which took 44 minutes (± 25 minutes). The shortest average duration was in phase 8, lasting 6 minutes (± 5 minutes).
A descriptive statistical analysis of all videos showed that each video starts with phase 1 and ends with phase 8. Every video has an occurrence of each phase at least once, except for the exceptional phase 0. Except for the start and end phases, the order of the phases might vary in the videos. In total 217 phase transitions were recorded, averaging 9 per case. The most common phases were phase 4, with an average duration of 16 minutes (± 17 minutes), and phase 5, with an average duration of 15 minutes (± 17 minutes). The least common phase was phase 0, which had an average duration of 34 minutes (± 30 minutes).

The performance of the ML algorithm showed an overall accuracy of 0.63 ± 0.08 and F1 score of 0.60 ± 0.09. The precision was at 0.68 ± 0.1, the recall at 0.63 ± 0.08 and the overlap score at 0.38 ± 0.17.

The highest F1 scores were for phase 2 (0.77 ± 0.19) and 7 (0.87 ± 0.10) and the lowest F1 scores were for phase 0 (0 ± 0.00), 4 (0.17 ± 0.17), 5 (0.22 ± 0.23) and 8 (0.31 ± 0.34).

**Conclusion:**
The study seeked to identify an approach to develop a ML model for phase recognition in right hemicolectomy. With the results we showed that with applying TeCNO we developed a model feasible to recognize phases in this specific colorectal procedure.
However, we need to investigate how to improve the performance regarding the phases which performed poorly potentially due to shorter phase length and resulting class imbalance. Furthermore, we wil investigate different machine learning models.

### Can Virtual Robotic Training Contribute To Current Robotic Education?

ID: 307

**Kategorie: DGCH - Robotische Forschung und KI**

Justus Baecker[1], Fenja Friederike Thom[2], Stephan Kersting[1], Richard Hummel[3]

[1]*Klinik für Allgemeine Chirurgie, Viszeral-, Thorax- und Gefäßchirurgie, Universitätsmedizin Greifswald, Greifswald, Deutschland*, [2]*Universitätsmedizin Greifswald, Klinik für Allgemeine Chirurgie, Viszeral-, Thorax- und Gefäßchirurgie, Greifswald, Deutschland*, [3]*Klinik für Allgemeine Chirurgie, Viszeral-, Thorax- und Gefäßchirurgie, Universitätsmedizin Greifswald , Greifswald, Deutschland*

**Background:**

Robotic surgery has become a critical component of modern clinical practice, offering enhanced safety without compromising surgical quality.[1] Given the rapid expansion of robotic-assisted procedures, and the increase in different procedures performed robotically, it is imperative to integrate residents into surgical programs and provide them with comprehensive training. As with any surgical skill, consistent practice and repetition are crucial to improving patient outcomes.[2]

However, the high cost of robotic systems, coupled with their extensive use during operating hours, limits their availability for training purposes, which raises the challenge of ensuring regular exposure to robot-assisted surgical training. For this reason, we advocate for the use of simulators that enable virtual robotic training as a viable alternative to traditional physical models. Simulators are not only significantly more cost-effective in the long term but also do not require operating room space.

**Materials and methods:**

This study aims to evaluate the effectiveness of virtual robotic training using a simulator compared to conventional robotic training with physical models. Sixteen medical students were randomized into two groups: one group performed five designated tasks using a simulator, while the control group completed the same tasks on a physical model. Both groups then undertook a final, more complex task—a gastroenterostomy—on a physical model. The outcomes measured included time to completion, number of errors, and GEARS (Global Evaluative Assessment of Robotic Skills) scores, with the goal of determining whether virtual training can effectively supplement traditional robotic training and improve training accessibility for surgical residents.

**Results:**

Preliminary findings indicate comparable learning outcomes between the group trained on robotic systems and the group trained using simulators. Both groups demonstrated similar proficiency across the assessed tasks. Final data analysis is ongoing, and conclusive results are pending.

**Conclusion:**

We anticipate that increasing the availability of training opportunities, particularly through the use of simulators, will enhance surgical proficiency. Expanding access to robotic training for young surgeons is expected to yield long-term improvements in surgical performance. Whether these outcomes can be fully achieved through the use of virtual models alone, or if physical models remain necessary, is a subject for further investigation.

1. Phan K, Kahlaee HR, Kim SH, Toh JWT. Laparoscopic vs. robotic rectal cancer surgery and the effect on conversion rates: a meta-analysis of randomized controlled trials and propensity-score-matched studies. Tech Coloproctol. 2019;23(3):221-230. doi:10.1007/s10151-018-1920-0
2. Azadi Shirin, Green Isabel C, Arnold Anne, Truong Mireille, Potts Jaqueline, Martino Martin A. Robotic Surgery: The Impact of Simulation and Other Innovative Platforms on Performance and Training. J Minim Invasive Gynecol. 2021 Mar;28(3):490-495. doi: 10.1016/j.jmig.2020.12.001

### Zero-shot Performance of Large Language Models for Differential Diagnosis in Acute Abdominal Pain

ID: 393

**Kategorie: DGCH - Robotische Forschung und KI**

Jonas Henn[1 2], Philipp Feodorovici[2 3], Simon Hatterscheidt[1 2], Alisa Stoll[1 2], Dhanakumaresh Subramani[2], Jonas Dohmen[1 2], Ingo Gräff[4], Jörg C. Kalff[1 2], Hanno Matthaei[1 2]

[1]*Klinik und Poliklinik für Allgemein-, Viszeral-, Thorax- und Gefäßchirurgie, Universitätsklinikum Bonn , Bonn, Deutschland*, [2]*Bonn Surgical Technology Center (BOSTER), Universitätsklinikum Bonn, Bonn, Deutschland*, [3]*Abteilung für Thoraxchirurgie, Klinik und Poliklinik für Allgemein-, Viszeral-, Thorax- und Gefäßchirurgie, Universitätsklinikum Bonn, Bonn, Deutschland*, [4]*Abteilung für Klinische Akut- und Notfallmedizin, Universitätsklinikum Bonn, Bonn, Deutschland*

**Background:**

Acute abdominal pain (AAP) is a common presenting symptom in the emergency department (ED), with a wide range of potential causes and diagnoses. Moreover, the mortality rate for AAP is markedly higher than that observed in patients presenting with chest pain and in many cases, a decision must be made regarding the necessity of emergency surgical care. A critical juncture in the treatment pathway is the accurate differential diagnosis, which enables the prompt initiation of targeted therapy. Recently, large language models (LLM) have been ascribed clinical reasoning abilities at physicians' level. The objective of our study is to examine the comparative performance of LLM in differential diagnosis in the context of AAP.

**Materials and methods:**

We retrospectively analyzed 100 cases of adult patients presenting with AAP to the ED. The final diagnosis according to the emergency report or the final discharge letter, if applicable, was set as ground truth. Case vignettes were presented to three publicly available chatbots (ChatGPT Plus, Gemini Advanced, and Le Chat) as well as medical students and board-certified abdominal surgeons. Accuracy of the primary differential diagnosis, the top three differential diagnoses, and the urgency classification was compared.

**Results:**

The study included 100 patients who presented with AAP at the emergency department of Bonn University Hospital. Of those, 52 were female and the median age was 49 years (IQR: 29.8-62.3). The most common diagnoses were non-specific abdominal pain (25 %), appendicitis (10 %), hernia (9 %), bowel obstruction / ileus (7 %), and cholecystitis (7 %). In the primary differential diagnosis, the surgeons outperformed the LLM, who then demonstrated superior performance compared to the students (Accuracy and 95CI: Surgeons: 0.44 (0.32-0.56), Gemini 0.42 (0.32-0.52), ChatGPT 0.38 (0.28-0.48), Le Chat 0.39 (0.29-0.49), Students 0.35 (0.26-0.44)). In evaluating the top three differential diagnoses, the LLMs demonstrated comparable accuracy to that of the surgeons, whereas the students exhibited a notable decline in performance (Accuracy and 95CI: Surgeons: 0.70 (0.59-0.81), Gemini 0.70 (0.61-0.79), ChatGPT 0.72 (0.65-0.83), Le Chat 0.48 (0.38-0.58), Students 0.55 (0.45-0.65)). In evaluating the urgency of the suspected diagnosis, the surgeons demonstrated a more accurate assessment than the LLM and the students (Accuracy and 95CI: Surgeons: 0.71 (0.60-0.82), Gemini 0.64 (0.55-0.73), ChatGPT 0.66 (0.57-0.75), Le Chat 0.66 (0.57-0.75), Students 0.62 (0.52-0.72)). In detail, the LLM demonstrated superior sensitivity (Surgeons 0.83, Gemini 0.98, ChatGPT 0.94, Le Chat 0.98, Students 0.82), whereas the human participants exhibited considerably elevated specificity (Surgeons 0.63, Gemini 0.31, ChatGPT 0.39, Le Chat 0.35, Students 0.43). The LLM rarely suspected non-urgent and simple diagnoses such as cholecystolithiasis and constipation. These were instead classified as cholecystitis and ileus / bowel obstruction respectively. In all cases where none of the LLM models correctly identified the urgency of a diagnosis, the underlying condition was, in fact, non-urgent.

**Conclusion:**
AAP represents a heterogeneous group of diagnoses, posing a significant clinical challenge even for experienced surgeons. Thus, in evaluating the LLM, it is essential to consider not only the absolute metrics but also the comparative performance. Medical students are the next generation of primary assessors in the ED. Here, LLM could provide decision support to these less experienced physicians on call. To illustrate, the physician may be prompted to comprehensively review the LLM's differential diagnosis and to compare it with his own evaluation. For example, in the case of the LLM suspecting an ileus, a rapidly performed computer tomography would be an appropriate diagnostic tool to prevent possible delays and hazards to the patient. Furthermore, the LLM differential diagnosis and urgency assessment in the ED could be employed prior to contacting the doctor, for instance, to determine the most appropriate specialist discipline for the case. The online LLMs naturally offer exceptional transferability, as the training was conducted on general data sets. Nevertheless, we will evaluate local LLM with the objective of achieving more transparency and, ultimately, also for data protection reasons.

**Automating Surgical Video Analysis and Documentation for Minimally Invasive Surgery with Vision Language Models: A Comparative Evaluation of GPT-4o and Gemini 1.5 Pro**
ID: 560

**Kategorie: DGCH - Robotische Forschung und KI**

Esther Stüker[1], Marko van Treeck[1], Fiona R Kolbinger[1 2 3], Florian Oehme[2 3], Marius Distler[2 3], Jakob Nikolas Kather[1 4 5], Hannah Sophie Muti[1 2 3]

[1]*Else Kröner Fresenius Center for Digital Health, Dresden University of Technology, Dresden, Germany, Dresden, Deutschland*, [2]*Department for Visceral, Thoracic and Vascular Surgery, University Hospital and Faculty of Medicine Carl Gustav Carus, Technische Universität Dresden, Dresden, Germany, Dresden, Deutschland*, [3]*National Center for Tumor Diseases Dresden (NCT/UCC), a partnership between DKFZ, Faculty of Medicine and University Hospital Carl Gustav Carus, TU Dresden University of Technology, and Helmholtz-Zentrum Dresden - Rossendorf (HZDR), Dresden, Germany, Dresden, Deutschland*, [4]*Medical Department 1, University Hospital and Faculty of Medicine Carl Gustav Carus, Technische Universität Dresden, Dresden, Germany, Dresden, Deutschland*, [5]*Department of Medical Oncology, National Center for Tumor Diseases (NCT), Heidelberg University Hospital, Heidelberg, Germany, Heidelberg, Deutschland*

**Background:**
Large Language Models (LLMs) have already demonstrated in numerous studies their potential as valuable support tools in the field of medicine. LLMs specifically designed for biomedical approaches have been shown to outperform physicians in a range of text-related medical tasks, such as exam question answering or medical documentation. With the advent of Large Language Models equipped with vision capabilities (vLLMs), the scope of potential applications in medicine has expanded further. However, a publicly available biomedical vLLM has not been released to date. Surgeons work with a wide range of image and video data from various diagnostic and interventional procedures daily. The amount of video data is constantly rising due to exponential use of Minimally Invasive Surgery (MIS) since its introduction. Each procedural information has to be condensed into a written report, which is repetitive and consumes a great amount of time. Integrating vLLMs into surgical documentation from image and video data could alleviate the documentation burden for surgeons, allowing more valuable time for direct patient care. Hence, we aimed to investigate whether vLLMs can perform intraoperative object detection and concept understanding, surgery type identification and the generation of complete and accurate surgical reports.

**Materials and methods:**
We used the publicly available CholecT45 dataset consisting of 45 videos of laparoscopic cholecystectomies (CCE), of which 15 videos were randomly selected. Frames were extracted at a rate of one frame per second and cropped to 856x480 in jpeg format**.** The video frames were analyzed using an end-to-end vLLM-based pipeline: First, the vLLM was instructed to perform surgical image analysis using a predefined system prompt, which emphasized that it must perform a surgical image analysis task while strictly adhering to the video content and avoiding to include any information not explicitly shown. Then, the video frames were provided to the vLLM together with a user prompt specifying the respective image analysis task. The surgical image analysis tasks included the detection of clips, retrieval bags and surgical cloths, the detection of active bleeding and the surgery type identification (in this case, CCE). For the generation of surgical reports, we created a template outlining the key steps of a CCE, which was then included in the system prompt as a blank template for the model to fill and as a complete text for comparison. This yielded two reports per video: one where the model autonomously completed the blank template and another one where it modified the filled template.
This experimental setup was followed with customized pipelines querying two different vLLMs , GPT-4o and Gemini 1.5 Pro, for comparison, with their respective Application Programming Interface (API). Descriptive statistics were used to quantify the model's accuracy for a specific task.

**Results:**
For clip detection, both GPT-4o and Gemini1.5Pro identified the presence of vessel clips in 100% of cases. The retrieval bag was correctly detected in 100% of cases by GPT-4o, while Gemini 1.5 Pro achieved 93.33% accuracy. For detecting the use of surgical cloths, GPT-4o was accurate 93.33% of the time, whereas Gemini1.5Pro reached only 60% accuracy. In detecting active bleeding, Gemini 1.5 Pro performed slightly better with 93.33% accuracy, compared to GPT-4o’s 86.67%. Gemini 1.5 Pro successfully identified the given videos as cholecystectomies 93% of the time, outperforming GPT-4o, which identified the type of surgery in 80% of all cases. For the generation of complete and accurate surgery reports, both models produced similar results on average. When an empty template was provided as part of the system prompt, GPT-4o generated reports that were, on average, 90% complete and 64% correct. In comparison, Gemini 1.5 Pro achieved an average of 88% completeness and 71% correctness for the same task. When working with a pre-filled template that required modification to accurately reflect the course of the surgery, GPT-4o generated reports that were, on average, 90% complete and 70% correct, while Gemini 1.5 Pro produced reports that were, on average, 91% complete and 71% correct.

**Conclusion:**
Despite a very simple experimental approach using a general vLLM without specification for biomedical tasks, both vLLMs performed surgical object detection, concept understanding, and surgery type identification with a high accuracy. The accuracy of surgical report writing, however, remains to be improved. Benchmarking these results against an upcoming vision- language model for surgery could be beneficial for further advancements. Moreover, models with more parameters and larger context windows are expected to be released in the near future, facilitating the analysis of large and complex medical datasets, as is surgical video data.

### The Rise of Robotic Assisted Microsurgery in Reconstructive Procedures: Prospects and Challenges

ID: 684

**Kategorie: DGCH - Robotische Forschung und KI**

Khaled Dastagir[1], Peter M. Vogt[1]

[1]*Medizinische Hochschule Hannover, Klinik für Plastische, Ästhetische, Hand- und Wiederherstellungschirurgie, Hannover, Deutschland*

**Background:**
Robotic-assisted microsurgery has emerged as a significant innovation in reconstructive surgery, offering enhanced precision, dexterity, and control that can improve surgical outcomes in complex microsurgical procedures, including nerve repairs and vascular anastomoses.

**Materials and methods:**
This prospective study evaluates the progression of a microsurgeon's skills through the analysis of 40 clinical applications of the Symani Surgical System, focusing on metrics such as anastomosis counts, times, and learning curves. Additionally, a case series involving 10 patients undergoing high-end surgeries for reconstructions of various anatomical sites was conducted.

**Results:**
The analysis of the 40 cases revealed a total of 120 anastomoses completed, with an average anastomosis time decreasing from 45 minutes in initial cases to 17 minutes by the end of the study period, indicating a significant improvement in efficiency. The learning curve demonstrated a reduction in time taken per anastomosis, suggesting that proficiency increased with experience.

**Conclusion:**
The findings highlight the potential of robotic-assisted microsurgery to enhance surgical outcomes through improved precision and reduced operative times. However, challenges such as the high cost of robotic systems and the steep learning curve for surgeons remain significant barriers to widespread adoption. Essential training protocols are necessary to facilitate integration into current surgical workflows. Continued research and development are imperative to establish robotic systems as standard tools in reconstructive microsurgery.

**Optimizing Large Language Models for Decision Support in Multidisciplinary Tumor Boards: A Feasibility Study LLM4Tumor**
ID: 798

**Kategorie: DGCH - Robotische Forschung und KI**

Cui Yang[1], Priska Hakenberg[1], Schaima Abdelhadi[1], Alida Finze[1], Melissa Harbrücker[1], Christoph Reißfelder[1]

[1]*Universitätsmedizin Mannheim, Chirurgische Klinik, Mannheim, Deutschland*

**Background:**
Multidisciplinary tumor boards are essential for developing optimal treatment strategies for oncological patients. Large Language Models (LLMs) such as GPT-4 and Llama show potential as supportive tools due to their ability to process vast amounts of clinical data. However, their direct clinical application is currently limited by lack of refinement for this specific use case. This study aims to evaluate the feasibility of optimizing LLMs to support decision-making within multidisciplinary tumor boards.

**Materials and methods:**
The study utilizes a structured dataset of tumor board cases from the past three years. The methodology includes: 1. Data preparation: Processing and cleaning of existing anonymized patient data. 2. Baseline evaluation: Comparing unoptimized LLM recommendations to actual tumor board decisions using a Likert scale. 3. Retrieval-augmented generation (RAG): Enhancing LLMs with up-to-date medical guidelines to improve recommendation accuracy and traceability. 4. Fine-tuning: Refining LLMs using 80% of the tumor board dataset to adapt them to the nuances of oncological decision-making in a high-volume center.

**Results:**
We anticipate demonstrating the potential of optimized LLMs in supporting tumor board decision-making processes. Expected outcomes include improvements in the accuracy, transparency, and traceability of AI-generated recommendations, potentially exceeding current medical guidelines by incorporating the latest advancements in oncology.

**Conclusion:**
This project aims to develop a proof of concept for an AI-based decision support tool that can enhance the quality and efficiency of tumor board recommendations. If successful, it could pave the way for the integration of optimized LLMs in clinical settings, ultimately improving patient care in oncology.

### Pilot Study on the Implementation of Artificial Intelligence in the Operating Room as a Replacement for Manual Closing Counts

ID: 843

**Kategorie: DGCH - Robotische Forschung und KI**

Simon Störzer[1], Felix Gronau[1], Ivan Delgadillo Aceves[1], Moritz Schmelzle[1], Maximilian Kaltenecker[2], Jacques Hahn[2], Geetha Mahadevappa[2], Gerrit Grannas[1]

[1]*Medizinische Hochschule Hannover (MHH) , Klinik für Allgemein-, Viszeral- und Transplantationschirurgie, Hannover, Deutschland*, [2]*Nexustec GmbH, München, Deutschland*

**Background:**
The operating room (OR) is a high-stakes environment where patient safety is paramount. One critical safety procedure is the manual closing count, performed by the OR staff at the conclusion of surgery to ensure that all instruments and materials are accounted for before the surgical wound is closed. This process, however, is susceptible to human error due to factors such as the duration of the surgery, the experience of the staff, and the complexity of the operation. Errors in this process can lead to serious complications, including retained surgical items (RSIs), which pose significant risks to patients. This pilot study aims to explore the application of artificial intelligence (AI) in replacing manual closing counts, with the goal of improving both accuracy and efficiency while enhancing patient safety.

**Materials and methods:**
The pilot study is designed to evaluate the use of AI systems developed by Nexustec GmbH, Munich, which utilizes camera-based technology to recognize and track surgical instruments and materials through various identifiers such as contour, shape, and color. The study will focus on the Accuracy of Counting. The primary goal was to assess how accurately the AI system can perform closing counts. The system's ability to recognize and count surgical tools and materials was analyzed in 100 runs.

**Results:**
Preliminary testing with Nexustec's AI system indicates promising results. The system was able to accurately recognize and count surgical instruments and materials based on contour, shape, and other identifiers, demonstrating a high level of precision. In the simulation trials, the AI system demonstrated a remarkable ability to recognize and count instruments of all types and sizes with 100% accuracy. This included surgical instruments of varying dimensions and complexity, as well as needles as small as 6-0, which were correctly identified in all cases. These results were consistent across all trials, providing strong evidence of the system's potential to eliminate human error during closing counts.

**Conclusion:**
This pilot study demonstrates the potential for AI to revolutionize the closing count process in the operating room. By improving accuracy, efficiency, and overall workflow, AI systems like the one developed by Nexustec GmbH offer a promising solution to a longstanding safety concern. While further testing and validation are required, the results of this pilot study indicate that AI could become a valuable tool in ensuring that surgical instruments and materials are properly accounted for, ultimately enhancing patient safety and reducing the risks associated with retained surgical items.

Abstracts – DCK 2025 – 142nd Congress of the German Society of Surgery – Munich, March 26–28 • DOI 10.1515/iss-2025-2002
Innov Surg Sci 2025; 10 (Special Suppl 1): s40–s112

**Implementation of a Multidisciplinary Single-Port Robotic Surgery Program at a Robotic Surgery Center**
ID: 853

**Kategorie: DGCH - Robotische Forschung und KI**

Justus Baecker[1], Stephan Kersting[1], Richard Hummel[1]

[1]*Universitätsmedizin Greifswald, Klinik und Poliklinik für Allgemeine Chirurgie, Viszeral-, Thorax- und Gefäßchirurgie, Greifswald, Deutschland*

**Background:**
Single-port surgery (SILS) is a minimally invasive technique that has seen limited adoption, despite its potential benefits, including reduced scarring, decreased postoperative pain, and shorter recovery times due to a single access point to the abdominal cavity. However, its usage has been limited by technical challenges, such as restricted instrument maneuverability and the need for advanced surgical skills.
The rise of robotic-assisted surgery and the widespread availability of robotic systems capable of performing complex procedures have further overshadowed SILS. With the introduction of the Single-Port (SP) robotic system, many of the handling difficulties associated with single-port instruments seem to be mitigated. Nevertheless, experience with SP robotic systems remains limited, and their use has thus far been restricted to a few specific procedures, such as transoral robotic surgery (TORS) and robotic prostatectomies.

**Materials and methods:**
We initiated a multidisciplinary SP robotic surgery program using a stepwise approach, which was previously published by our team. This program was implemented across four specialties: general surgery, thoracic surgery, urology, and otorhinolaryngology. The rollout began with lower-complexity procedures, such as cholecystectomies, with plans to progressively increase the complexity of the cases as surgical teams gained experience with the SP system.
To ensure a smooth transition, only surgeons, table assistants, and operating room (OR) nurses with prior experience using the Xi robotic system were included in the initial phase of the SP program. For cases where lower-complexity surgeries were not feasible, external proctors with expertise in SP systems were invited to support the training and facilitate the rollout.
Data were collected on several key performance indicators, including OR time, surgeon learning curves, postoperative complications (classified using the Clavien-Dindo grading system), length of hospital stay, ICU admission and duration, as well as training time required for surgeons to become proficient in SP surgery.

**Results:**
Preliminary data suggest that SP robotic surgery shows promise in reducing surgeon training times and facilitating the transfer of skills for surgeons already experienced in robotic surgery. Compared to traditional single-port laparoscopic surgery, many of the limitations—such as reduced maneuverability—are significantly minimized with the SP robotic system. However, additional time and experience are needed to fully assess the system's impact on surgical outcomes and efficiency.
Early results show acceptable operative times and low complication rates, particularly in low-complexity cases. Surgeons have reported a shorter learning curve for SP robotic procedures compared to the initial learning curve experienced during their primary robotic training. Nonetheless, the program is still in its infancy, and more data are required to draw definitive conclusions regarding the system's long-term benefits.

**Conclusion:**
The introduction of single-port robotic surgery represents a promising advancement in the field of minimally invasive surgery, offering the potential for even smaller incisions and faster patient recovery. While SP surgery requires additional training for robotic surgeons, preliminary evidence suggests that the learning curve is significantly shorter than for initial robotic training.
The full extent of procedures that can be performed using the SP system, particularly in more complex cases and new surgical fields such as retroperitoneal surgery, remains to be explored. Long-term data comparing the outcomes and benefits of SP surgery to those of multi-port robotic surgery are needed to fully validate the role of SP systems in the future of robotic surgery.

**Does previous robotic surgical experience impact on implementation of new robotic system?**
ID: 855

**Kategorie: DGCH - Robotische Forschung und KI**

Justus Baecker[1], Stephan Kersting[1], Richard Hummel[1]

[1]*Universitätsmedizin Greifswald, Klinik und Poliklinik für Allgemeine Chirurgie, Viszeral-, Thorax- und Gefäßchirurgie, Greifswald, Deutschland*

**Background:**
The introduction of the Single-Port (SP) robotic system has the potential to revolutionize robotic surgery by addressing some of the technical challenges previously associated with single-port instruments in Single-Incision Laparoscopic Surgery (SILS). Although early data suggest improvements in handling, the use of SP robotic systems remains limited, with applications thus far confined primarily to transoral robotic surgery (TORS) and robotic prostatectomies.
In contrast, the multi-port robotic surgery platform, such as the Da Vinci Xi system, has become well-established across various specialties, including general surgery, urology, and gynecology. Transitioning to a new system like the SP, with unexplored applications and different technical limitations, presents a unique set of challenges. Our current study aims to compare the difficulties encountered during the rollout of the Xi system with those experienced during the implementation of the SP system, while assessing whether prior experience with the Xi system eases the transition.

**Materials and methods:**
Two years ago, we launched a robotic surgery program using the Xi system. During this time, we collected comprehensive data on key performance indicators (KPIs), including:

- Operating room (OR) time, including preparation time,
- Surgeon learning curves,
- Postoperative complications, classified using the Clavien-Dindo grading system,
- Length of hospital stay,
- ICU admission rates and duration.

At the outset, most of the in-house surgeons had limited experience with robotic surgery. We adopted a stepwise approach, initially performing lower-complexity procedures such as cholecystectomies and inguinal hernia repairs, and gradually progressing to more complex cases as the surgical teams became more proficient.
With the rollout of the SP robotic system, we used a similar stepwise approach. The same KPIs were tracked for comparison. However, this time, we expanded the program to include additional specialties such as thoracic surgery and otorhinolaryngology, thereby increasing the diversity of procedures performed. We also documented learning curves for surgeons transitioning from the Xi to the SP system, as well as for those new to robotic surgery in different surgical fields.

**Results:**
Preliminary data suggest that prior experience with the Xi system significantly reduces surgeon training times and facilitates the transfer of core skills to the SP system. Surgeons with established robotic experience demonstrated faster adaptation to the new platform compared to those starting their robotic training afresh. Despite this, initial challenges related to reduced instrument maneuverability within the limited working space of the SP system persisted. However, simplified OR setup and reduced preparation times helped offset some of these limitations.
Early results show that operative times and complication rates are comparable between the Xi and SP systems, particularly in lower-complexity cases. Surgeons reported that the learning curve for SP procedures was shorter than the initial learning curve encountered during their early robotic training with the Xi system. Nonetheless, intraoperative handling was perceived as more complex in SP procedures due to the reduced workspace and the need for precise instrument coordination.
Although the program is still in its early stages, these preliminary findings indicate that while SP robotic surgery presents distinct technical challenges, the learning curve for experienced robotic surgeons is notably shorter.

**Conclusion:**
The introduction of the SP robotic system represents a significant advancement in minimally invasive surgery, offering potential benefits such as a streamlined robotic setup and reduced preparation time. However, the reduced working space introduces new intraoperative complexities that require additional training and skill refinement.
Our study demonstrates that previous experience with the Xi robotic system plays a crucial role in easing the transition to SP robotic surgery. Surgeons with prior robotic experience benefit from shorter learning curves, and their familiarity with robotic principles allows for smoother adaptation to the new system. Establishing a new robotic platform like the SP system can thus be significantly facilitated by prior expertise in other robotic systems, contributing to the overall success of its implementation.
Long-term data are still needed to fully evaluate the clinical and operational benefits of the SP system compared to the multi-port Xi system, particularly in more complex and diverse surgical cases.

**Comparison of Robotic-Assisted Perforator-to-Perforator DIEP Flaps vs. Robotic and Conventional DIEP Flaps: a retrospective cohort study.**
ID: 908

**Kategorie: DGCH - Robotische Forschung und KI**

Laetitia Chiarella[1 2], Kai Wessel[1 2], Charalampos Varnava[1 2], Tobias Hirsch[1 2], Maximilian Kückelhaus[1 2]

[1]*Department of Plastic and Reconstructive Surgery, Institute for Musculoskeletal Medicine, Münster, Deutschland*, [2]*Department of Plastic, Reconstructive and Aesthetic Surgery, Hand Surgery, Fachklinik Hornheide,, Münster, Deutschland*

**Background:**
Deep Inferior Epigastric Perforator (DIEP) flap breast reconstruction has become standard of care following mastectomy, offering the advantage of transferring autologous tissue while minimizing donor site morbidity. (1,2) Traditional DIEP procedures involve the microvascular anastomosis of the deep inferior epigastric vessels to the internal mammary artery and vein (IMA). Recent advancements in supermicrosurgery and robotic assistance, particularly the Symani Surgical System, have facilitated precision in complex procedures like perforator-to-perforator anastomosis, improving ergonomics and potentially enhancing outcomes. (3) However, there is limited comparative data regarding traditional DIEP flap reconstruction versus robotic-assisted techniques. This study aims to compare the outcomes of conventional DIEP flap reconstruction with robotic-assisted microsurgery and robotic-assisted perforator-to-perforator anastomosis, focusing on surgical complications, hospital length of stay (HLOS), and postoperative pain management.

**Materials and methods:**
This single-center retrospective cohort study included 91 patients who underwent DIEP flap breast reconstruction between January 2022 and July 2024. Patients were divided into three groups based on the type of surgery: conventional (n=51), robotic-assisted (n=28), and robotic-assisted perforator-to-perforator anastomosis (n=12). Patient demographics, surgical complications, HLOS, and postoperative pain management were analyzed. Surgical complications were classified as major or minor using the Clavien-Dindo classification, and pain control was assessed based on the postoperative use of piritramide and patient-reported Numeric Rating Scale (NRS) pain scores.
Statistical analyses included multivariable logistic regression for surgical complications and linear regression for HLOS and postoperative pain medication use. Adjusted odds ratios (aOR), adjusted risk differences (aARD) and 95% confidence intervals (CI) are presented for multivariable logistic and linear regression models. Propensity score matching (PSM) was used to control for confounding variables such as age and BMI. Additionally, exploratory Poisson regression analysis was conducted to examine the incidence rate of complications across the surgical groups.

**Results:**
The study population were on average 50.44 ± 10.56 years old and presented with a mean BMI of 27.16 ± 4.30. The majority of patients (85.71%) had an American Society of Anesthesiologists (ASA) status of 2. Patients in the robotic-assisted perforator-to-perforator group were significantly younger than those in other groups (P=0.018). Arterial hypertension was more prevalent among patients in the robotic-assisted group (P=0.006), but no significant differences in BMI or other comorbidities were observed between groups.
A total of 29.67% of patients experienced surgical complications, with 18.52% classified as major. Complication rates were lower in the robotic-assisted (25.00%) and perforator-to-perforator groups (8.00%) compared to the conventional group (37.25%), though these differences were not statistically significant (P > 0.05). The adjusted odds ratio (aOR) for surgical complications in the robotic-assisted group was 0.23 (95% CI, 0.02–2.29; P=0.21), and for the perforator-to-perforator group, it was 0.46 (95% CI, 0.13–1.63; P=0.23).
The average HLOS was similar across all groups, with no significant differences observed after adjustment for confounders (P > 0.05). However, patients in the perforator-to-perforator group required significantly less postoperative piritramide for pain management compared to the conventional group (adjusted average risk difference [aARD] -9.61 mg; 95% CI, -15.75 to -3.47; P=0.003). Postoperative NRS pain scores were also lower in the perforator-to-perforator group, but the differences were not statistically significant (P=0.60).
Propensity score matching confirmed the significant reduction in piritramide use in the perforator-to-perforator group, with an estimated average treatment effect (ATE) of -6.22 mg (P=0.01). In the exploratory Poisson regression analysis, patients in the perforator-to-perforator group had a significantly lower incidence rate of complications compared to those undergoing conventional surgery (P=0.020), although this finding lost statistical significance after adjusting for confounders (P=0.051).

**Conclusion:**
Robotic-assisted perforator-to-perforator anastomosis in DIEP flap breast reconstruction is non-inferior to conventional techniques in terms of surgical complications and HLOS. The most notable advantage of the robotic-assisted perforator-to-perforator technique was a significant reduction in postoperative pain medication requirements, suggesting enhanced pain control for patients. While these findings are promising, further prospective studies are warranted to evaluate long-term outcomes, patient satisfaction, and the broader applicability of robotic-assisted techniques in breast reconstruction.

**References**

1. Haddock NT, Teotia SS. Efficient DIEP Flap: Bilateral Breast Reconstruction in Less Than Four Hours. *Plast Reconstr Surg Glob Open*. 2021;9(9):e3801. doi:10.1097/GOX.0000000000003801
2. Varnava C, Klietz ML, Hirsch T, Wiebringhaus P, Kueckelhaus M. Comparison of surgical and patient-reported outcomes between 85 profunda artery perforator flaps and 122 deep inferior epigastric perforator flaps as first-choice treatment for breast reconstruction. *J Plast Reconstr Aesthetic Surg JPRAS*. 2023;80:168-177. doi:10.1016/j.bjps.2023.02.018

3. Wessel KJ, Wendenburg I, Gorji S, et al. Combined Application of a Novel Robotic System and Exoscope for Microsurgical Anastomoses: Preclinical Performance. *J Reconstr Microsurg Open*. 2023;08(02):e88-e96. doi:10.1055/a-2199-2584

### Towards AI-assisted complication prediction in robot-assisted minimally invasive esophagectomy: a prospective validation study on surgomic features

ID: 920

**Kategorie: DGCH - Robotische Forschung und KI**

André Schulze[1 2], Antonia Kraneis[1], Alexander Jenke[3 4], Johanna Brandenburg[1 2], Sebastian Bodenstedt[2 3 4], Rosa Klotz[5 6], Marius Distler[1], Jürgen Weitz[1], Stefanie Speidel[2 3 4], Martin Wagner[1 2]

[1]*Department of Visceral-, Thoracic- and Vascular Surgery, University Hospital Carl Gustav Carus, Technical University Dresden, Dresden, Deutschland*, [2]*Center for the Tactile Internet with Human in the Loop (CeTI), Technical University Dresden, Dresden, Deutschland*, [3]*Department for Translational Surgical Oncology, National Center for Tumor Diseases (NCT/UCC), Dresden, Deutschland*, [4]*Helmholtz-Zentrum Dresden-Rossendorf (HZDR), Dresden, Deutschland*, [5]*Department for General, Visceral, and Transplantation Surgery, Heidelberg University Hospital, Heidelberg, Deutschland*, [6]*The Study Centre of the German Surgical Society, Heidelberg University Hospital, Heidelberg, Deutschland*

**Background:**
Robot assisted minimally invasive esophagectomy (RAMIE) is a complex surgical procedure, after which a variety of life-threatening complications can occur. With Surgomics, we aim to predict the patient’s surgical outcome, by automatically extracting intraoperative features from the operation video by means of artificial intelligence (AI). In a previous study, two machine-learning algorithms were developed retrospectively to detect 10 frame-based surgomic features (bloodiness, smokiness, instrument, and anatomy presence) and 10 video-based surgomic features (phases of the procedure). We now aimed to validate the algorithms’ performance in a prospective study.

**Materials and methods:**
This is a multicentre prospective validation study performed at two University Hospitals Heidelberg and Dresden. We aimed to recruit 25 patients in each centre for a total of 50 patients. After written informed consent, we collected data along the treatment path of the patient pre-, intra-, and postoperatively including e.g. tumour biology and comorbidities. The postoperative data was collected through follow-up visits. Here, data regarding the postoperative complications and revision surgery was collected.
The two algorithms that are validated in this study are firstly a neural network that recognizes the above mentioned 10 frame-based surgomic features. Secondly, a Temporal Convolutional Network for the Operating room (TeCNO) to detect the 10 phases in the procedure. The annotations were done by at least two of a group of in total three medical experts with discrepancies being solved in discussion. For the feature annotation one frame every two minutes was equidistantly sampled and manually annotated regarding blood, smoke, two anatomical structures, and six instruments. The bloodiness in each frame was annotated on a scale from 0 to 4 and smokiness was annotated on a scale from 0 to 3. The remaining feature was annotated with a yes-or-no-tag describing its presence in the frame. The phase annotation was based on the temporal context of the video, meaning that the time of the beginning of each phase was annotated. In our definition, the RAMIE procedure consists of 10 phases, 5 phases in the abdominal part and 5 phases in the thoracic part of the surgery.

**Results:**
Final results are pending, as recruiting is still ongoing. Up until now 23 patients have been recruited. The Cohen’s kappa score for the annotated phases showed a very good agreement between the two annotators of 0.952. On average (+/- SD), the RAMIE procedure was 312±31 minutes long.
Regarding phase length, phase 5 “Completion of abdominal part” as well as phase 10 “Completion of thoracic part” were the shortest phases with a mean of 3.36±0.56 and 10.24±2.34 minutes. The longest phases were phase 2 “Gastric and distal oesophagus mobilisation” with 53.11±10.23 minutes and phase 7 “Dissection of the oesophagus” with 83.36±9.48 minutes. For the surgomic features, as of now annotations have been performed for 23 patients as well. In total 575 tags were used per video. The most frequently used tag was “Smoke-0” with about 136, followed by “Blood-2” with 87 tags per video. “Vessel Sealer” was used 44 times, “Azygos Vein” 43, “Permanent Cautery Hook” 30, “Gastric Tube” 26, “Blood-0” 26 and “Blood-3” 15 times per video.
The mean F1 score for AI-based phase recognition with TeCNO for the currently collected videos was 0.56±0.34 with a score of 0.59±0.32 for Dresden and 0.54±0.37 for Heidelberg. Phases that were particularly well recognized were “Preparation for abdominal part”, “Gastric and distal esophagus mobilization”, “Gastric tube construction”, “Preparation for thoracic part”, and “Dissection of the esophagus”. Poorly recognized phases were “Completion of abdominal lymphadenectomy”, “Completion of the abdominal part” and “Completion of the thoracic part”.
There was a significant difference in the F1 score between the centres for the phases “Completion of abdominal lymphadenectomy” and “Completion of abdominal Part” with Heidelberg-videos scoring a higher value than Dreden. For the phases“Dissection of esophagus” and “Insertion of anvil and stomach pull-up” Dresden-videos scored a higher value than Heidelberg.
Regarding the surgomic features the mean F1 score was 0.90±0.09 with a score of 0.91±0.08 for Dresden and 0.89±0.12 for Heidelberg. The features with the best score were “Large clip applier (Hem-O-Lok)” with 0.99 and “Clip applier (metal)” with 0.99. The overall F1 score for blood was 0.68 and for smoke 0.89.

**Conclusion:**
This abstract describes the first results of our ongoing clinical trial of a prospective validation of machine learning algorithms for complication prediction in RAMIE for esophageal cancer. Next steps include completion of patient recruiting and calculation of a predictive score for complications including surgomic features.

### Matrix Rib Fixation in Morbidly Obese COPD Patients: A Superior Alternative to Stracos Plates for Rib Fracture Stabilization

ID: 379

**Kategorie: DGCH - Sichere Chirurgie in allen Lebensaltern**

Varun Gupta[1], Lucia Cordes[1], Stefan Welter[1]

[1]*Lung Clinic Hemer, Department of Thoracic Surgery, Hemer, Deutschland*

**Background:**
Patients with significant comorbidities such as morbid obesity and chronic obstructive pulmonary disease (COPD), present unique challenges in thoracic surgery, especially in the management of rib fractures. We present the case of a patient with complex comorbidities and rib fractures, where a transition from Stracos to Matrix Rib fixation became necessary following repeated plate failure.

**Materials and methods:**
A 64-year-old male patient with a BMI of 38 presented with a primary diagnosis of left-sided hemothorax and multiple displaced rib fractures (C6, C7, and C8) following a traumatic incident. The patient's comorbidities included COPD Stage IV, chronic cough, arterial hypertension, diabetes mellitus, and bilateral leg lymphedema. Additionally, the patient had a history of intermittent atrial fibrillation managed with Eliquis, and prior groin hernia repair.
The initial surgery in April 2024 involved video-assisted thoracoscopic surgery (VATS) with conversion to thoracotomy for hematoma evacuation, debridement, partial pleurectomy, and stabilization of the C6, C7, and C8 rib fractures using one standard Stracos 9 segment plate and two Stracos 13 segment plates respectively. Two 24-Charriere chest drains and a Redon drain were placed. On the 9th postoperative day, the patient required re-thoracotomy due to spontaneous plate failure at C7 and C8 without external trauma. The broken plates were replaced with two standard Stracos 9 segment plates. The muscle layer was closed, and the patient received vacuum-assisted therapy (VAC) for the subcutaneous layers, with one VAC replacement on 5th postoperative day and secondary wound closure on the 8th postoperative day.
On the 12th postoperative day a second re-thoracotomy was required due to atraumatic breakage of the Stracos plates at C6 and C7. All Stracos plates were removed, and a new segment fracture at C8, with underlying necrosis, was identified and removed. Osteosynthesis was performed with Matrix Rib segment plates (10, 12, and 14) for C6, C7, and C8, respectively. As the thoracic cavity remained unopened during this procedure, only a 16-Charriere Redon drain was placed, without a chest tube.

**Results:**
The patient experienced recurrent Stracos plate failure, necessitating a change to Matrix Rib fixation. Post-operatively, there were significant improvements in respiratory function and mobility. A reduction in body weight from 138 kg to 128 kg was achieved, in part through loop diuretics, which also led to a decrease in bilateral leg edema and improved mobility. Despite initial challenges, the patient’s overall functional status improved, with reduced oxygen dependence, and significant enhancement in endurance.
Imaging conducted during the rehabilitation phase revealed no new fractures, plate dislocations or further material failures after Matrix Rib fixation. The wound showed signs of good healing.

**Conclusion:**
While the Stracos system offers advantages such as ease of use, reduced surgical time, decreased pain, and adaptability to rib anatomy, this case underscores its limitations in patients with severe obesity, advanced COPD, and chronic coughing, which strain the stabilization system. Matrix Rib fixation provided superior rib stability in this patient, reducing the risk of recurrent fractures and improving the overall clinical outcome. Multimodal rehabilitation played a key role in restoring the patient’s functional capacity.

### Outcomes of Lung Cancer Surgery in Elderly Patients: Age Group Comparison and the Role of Comorbidities

ID: 443

**Kategorie: DGCH - Sichere Chirurgie in allen Lebensaltern**

Fuad Damirov[1], Javad Karimbayli[2], Mircea Gabriel Stoleriu[3], Ughur Aghamaliyev[4], Gerhard Preissler[5]

[1]*Robert-Bosch-Krankenhaus, Schillerhöhe Lungenklinik, Abteilung für Thoraxchirurgie, Stuttgart, Deutschland*, [2]*IRCCS, National Cancer Institute, Aviano, Italien*, [3]*Asklepios Lungenklinik Gauting, Gauting, Deutschland*, [4]*LMU Klinikum, München, Deutschland*, [5]*Robert-Bosch-Krankenhaus, Stuttgart, Deutschland*

**Background:**
The prevalence of lung cancer and other tumors is increasing among the older adults. The aim of this study was to examine the influence of advanced age on the direct perioperative outcome as well as to identify potential risk factors that may lead to increasing morbidity and mortality after anatomical lung resections due to lung cancer in the era of VATS and ERAS.

**Materials and methods:**
A retrospective cohort study of the data of 180 older adults (141 >= 70 years, 39 >= 80 years) undergoing pulmonary anatomical resection for two most types of lung cancer from January 2019 to December 2023 was conducted. The patients were divided into two groups: septuagenarians and octogenarians. The risk of developing postoperative complications in association with the numerous observed factors, which appeared significant in univariate analysis, was assessed using univariate and multivariate logistic regression analyses. Readmission rate and mortality within 30 days were recorded.

**Results:**
There were 110 (61.2 %) men and 70 (38.2 %) women. 136 patients presented lung adenocarcinoma and 44 squamous cell carcinoma. A total of 17 segmentectomies, 154 lobectomies, 7 bilobectomies, and 2 pneumonectomies were performed through 125 VATS and 55 thoracotomy approaches. Readmission rate in septuagenarians and in octogenarians was 14.1 % and 25.6 % respectively. There was no difference in postoperative morbidity among the groups according to their age. According to multivariate logistic regression, longer duration of drainage treatment and longer stay on IMC unite appeared to be predictors for the development of complications.

**Conclusion:**
In our cohort, age more than 80 years was not found to be significant for the development of complications, when compared to the septuagenarians. The longer duration of drainage treatment and longer stay on IMC unite were significantly associated with the development of postoperative complications.

**Metabolic-bariatric surgery in patients above 65 years – a comprehensive report on national and regional trends regarding patient selection, procedure selection, complications, and results in the national StuDoQ cohort of 81.560 patients**
ID: 958

**Kategorie: DGCH - Sichere Chirurgie in allen Lebensaltern**

Stephan Herrmann[1], Lars Schmitt[1], Laura Matuschik[1], Karl-Peter Rheinwalt[2], Sylvia Weiner[3], Geoffroy Andrieux[4], Goran Marjanovic[1], Gabriel Seifert[1]

[1]*Universitätsklinikum Freiburg, Department Chirurgie, Allgemein- und Viszeralchirurgie, Freiburg, Deutschland*, [2]*Cellitinnen-Krankenhaus St. Franziskus, Adipositas-, Metabolische und Plastische Chirurgie, Köln-Ehrenfeld, Deutschland*, [3]*Sana Klinikum Offenbach, Adipositas Chirurgie und Metabolische Chirurgie, Offenbach, Deutschland*, [4]*Universitätsklinikum Freiburg, Institut für Medizinische Bioinformatik und Systemmedizin, Freiburg, Deutschland*

**Background:**
In the last issue of the national guidelines for metabolic-bariatric surgery (MBS), patients above 65 years of age (>65) were defined as a discrete cohort of patients with specific risk. The authors of the guideline recommended conducting these surgeries at certified centers. A first report with an exploratory data analysis (EDA) was presented at the German Viszeralmedizin 2024. Here, we aim to provide a comprehensive national StuDoQ registry report on MBS in patients >65.

**Materials and methods:**
In September 2024, we received a data export of the StuDoQ MBS registry. The dataset was curated prior to exploratory data analysis. We describe the demographic, anthropometric, medical features in patients >65, as well as the surgical procedures, postoperative complications and surgical results. We compare these results with patients below 65 years of age. Finally, we provide a model to estimate the adjusted difference in surgical results associated with age above 65.

**Results:**
The cohort includes 81.560 patients in total. 2.367 patients were above 65 years of age. The rate of surgery on a national level has increased annually during the last 8 years. On the level of participating states, there were considerable differences regarding age structure and MBS in >65. Employment status, ASA-score, age at onset of obesity, height, BMI, HbA1c, were significantly different between the groups. Procedural selection showed similar trends as in patients below 65 years of age and were congruent with guideline recommendations. >65 is significantly associated with less invasive, endoscopic procedures. Clavien-Dindo complications 2, 3a, 3b were more frequent in >65. Overall frequency of complications was comparable. Mortality showed no significant association with >65. Total Weight Loss (TWL) and remission of Type 2 Diabetes (T2D) differed significantly between the groups. At 2-3 years follow-up, TWL was 6% lower in >65.

**Conclusion:**
In clinical experience, patients >65 years of age present with more comorbidity. MBS indication warrants careful presurgical counselling, and results are not as pronounced as in younger patients. However, in this report we demonstrate that MBS is a safe and important therapy modality in patients >65 years of age with severe obesity and T2D with low complication rates and acceptable results. This report provides a representative and helpful estimate of MBS in patients >65 years of age for clinicians in the field.
